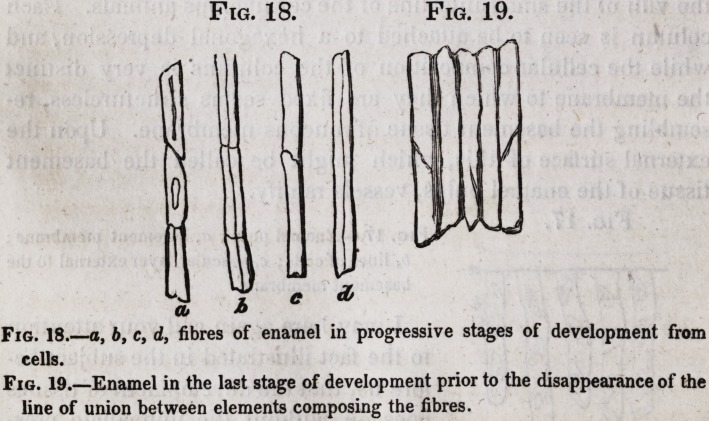# Lectures on Dental Physiology and Surgery, Delivered at the Middlesex Hospital School

**Published:** 1846-09

**Authors:** John Tomes

**Affiliations:** Surgeon Dentist to the Hospital.


THE AMERICAN

### Journal of Dental Science.

Vol. VII.] SEPTEMBER, 1846. [No. 1.

### ARTICLE I.

*A Course of Lectures on Dental Physiology and Surgery, delivered at the Middlesex Hospital School, Session* 1845–46. Expressly corrected for this Periodical by the Author, John Tomes, Esq., Surgeon Dentist to the Hospital.

### LECTURE I.

Gentlemen:—As dentist to the hospital of which you are pupils, it becomes my duty to give you some account of the cases which come under my care; to describe the nature of the diseases to which the teeth are liable, and the principles upon which they are most successfully treated. In doing so, it will be necessary for me to recal to your recollection the external form and situation of teeth; but in this I shall chiefly confine my observation to the human teeth; then the peculiarities of dental structure and development must be considered. Yet some anatomical points will be more intelligible by a comparison with other structures, or to the same structures as they occur in animals holding a lower place than man in the scale of organized beings. When we come to these, I shall go so far into the subject of comparative anatomy of the teeth as shall be subservient to our explanatory purpose.

Having considered the descriptive and structural anatomy of the dental apparatus, we shall pass to the main subject of these lectures—the diseases of the teeth and their treatment. The diseases of the temporary teeth will form the first division of the subject, while the maladies of the permanent teeth will form the second.

In children teething, with the diseases arising from interrupted dentition, are the most common, and at the same time the most formidable to which they are liable ; indeed, on reference to the bills of mortality of this metropolis, it will be seen that of those who die under the age of fifteen, four per cent. die from teething. This will at once show that too much attention cannot be given to dentition and its coincident disorders, by those engaged in medical studies. Many of these diseases will be treated of by your lecturer on midwifery, but dentition itself, with some of the symptoms of interrupted dentition, will necessarily come under our consideration.

The diseases of the permanent teeth are important from their frequency, and their painful character when present; and though not in themselves often dangerous to life, yet they exercise an important influence on other diseases situated in their neighborhood; as well as on indigestion and the various forms of nervous and spasmodic disease. During the year of 1814, of the 9140 out-patients who applied for relief at this hospital, 1050 applied with diseases of the permanent teeth. Thus, while the frequency of the disease commands our attention, it at the same time renders ignorance of the treatment unpardonable.

The last subject to engage our attention will be the means we possess of supplying the loss of the permanent teeth. Under this head will be described the construction of artificial teeth, the manner of using them, together with such information as the general practitioner may find useful when applied to by his patients for information upon the subject of artificial teeth.

Teeth may be defined to be hard bodies projecting from the surface of the mucous membrane, and situated in the alimentary canal, anterior to the pyloric orifice of the stomach, their use being subservient to nutrition. The hardness and density of the teeth bear a strict relation to the density of the skeleton, and it will be shown that the structure of the teeth, with few exceptions, holds an equally exact relation to the structure of the tissue composing the skeleton. In the class mammalia, the dental and osseous tissues reach their highest point of development and average density, while in fishes they are lower in organization and less hard; the structural relations being in this closer even than in the former class.

As regards situation, the teeth are placed in the alimentary canal, anterior to the pyloius, and are found to be occupying various part of this line, in strict conformity to the wants of the particular animal.

In animals as low in the scale as the *monads*, elementary teeth are found disposed around the mouth in the form of stiff spines. Among the *annelidæ* the leech is remarkable for sharp teeth, with which it inflicts its tri-radiate wound. The individual extremely minute teeth are sharp-pointed cones, fixed in single lines upon three semicircular-edged maxillæ, like the teeth of a circular saw. The maxillæ have their armed edges directed towards a common centre, and are each by suitable muscles moved upon their axes, and are at the same time made to approach each other; by this combination of movements, the animal saws the three lined wound with which we are so familiar.

In many insects, hard horny plates occupy the oral orifice, and perform the office of incisor teeth, while the œsophagus is provided with short, strong teeth, which perform the function of molars, as instanced in the grasshopper and others. The proventriculus in all mandibulated insects is armed with numerous horny teeth, which vary in form, number and arrangement, in the different species; some being flat, others corrugated on the surface, and some conical and recurved.

In structure the teeth of insects are similar to the external skeleton.*

In the division *mollusca*, of which the common snail is a member, we have a flat curved plate for incising, with which the creature divides the vegetable matter upon which it feeds. In the same order, some of the vegetable-feeding species are furnished with dental organs in the stomach.

In *fishes* the teeth are implanted in the vomer, in the palate, and pharyngeal bones, in the branchial arches and os hyoides, as well as in the maxillary and inter-maxillary bones.

* Burmister's Entomology.

In the *mammalians* the dental organs are alone found arranged in single lines in the maxillary and inter-maxillary bones.

Thus we find, by examining various members of the animal kingdom, teeth occupying every point of the anterior part of the alimentary canal.

Teeth are used either as organs for the mastication or prehension of food. They are also used as organs of offence and defence, and sometimes aid slightly in locomotion, and in man assist in articulation; but these may be considered as accessory uses only.

It admits of question whether the bills of birds should be regarded as dental organs; my opinion is that they should, since they are situated at the anterior orifice of the alimentary canal, are distinctly organs of prehension, and much used in the laceration of food, as instanced in carnivorous birds.

It may be objected that the structure is dissimilar to that of the skeleton; this objection will not, however, hold good, as we find in some animals the teeth have a structure similar to the cuticular appendages; as the nails such are the teeth of the ornithorhyncus. It becomes obvious, therefore, that they are two classes of teeth—osseous teeth, and cuticular teeth; the former having gelatine as their animal base, the latter albumen.* The structure of osseous teeth is similar to the structure composing the skeleton, whereas the cuticular teeth have a structure similar to the appendages of the epidermis. The transition from the one class to the other is not, however, sudden; for, although we do not find a mixture of the two structures in any tooth, yet we do find that the osseous teeth of some animals have properties in common with the cuticular teeth. Thus the incisor teeth of the rodentia grows during the whole life of the animal, and the cutting edge becomes extra-vascular from the recedence of its tubuli from their connection with the pulp cavity; a condition common to cuticular teeth, which are constantly increasing at the base, and are extra-vascular. Indeed, these ever-growing teeth may, in many respects, save in situation, be compared to the nails and horns. From these considerations, we must admit

* Owen's Odontography.

the bills of birds and of tortoises as belonging to the dental system.

A further confirmation of this opinion will be found in the manner of development, as instanced in the mandible of the parrot and tortoise. In the order of birds called Merganser, the mandibles are armed with conical sharp teeth.

In the jaw of the balæna mysticetus, we have the whalebone developed in the place of teeth; the structure of which is somewhat similar to that of the mandibles of birds.

By making this distinction in the two kinds of teeth, we are able to understand the conflicting opinions that existed so many years, but which recent investigation has set at rest; namely, as to whether the teeth were to be regarded as possessed of life and organization, or as inorganic excretions like the hair and nails.

Teeth admit of a general classification based upon their various external forms, and the purposes for which they are adapted. 1st. Teeth of a conical shape, with sharp points: such as are found in the canine teeth of the carnivorous animals, also in the more simple conical and lancet-shaped teeth of the shark tribe. Teeth of this class interlock with those of the opposing jaw, and are used either for seizing or retaining the prey. 2d. Teeth with sharped chisel-shaped edges for cutting, as the incisors of the rodentia, illustrated in the rabbit and rat. 3d. Teeth for tearing, lacerating, as the molars of carnivora. 4th. Teeth for crushing, as seen in the monkey tribes, where the principal food consists of fruits. 5th. Teeth for grinding: these teeth present a large flat surface, and the jaw is so articulated as to admit of great lateral motion. The grinders of the elephant afford an excellent example. This class of teeth belong mainly to animals subsisting on grain; which requires to to be reduced to powder before it is received by the organs of digestion. The conical-pointed tooth for piercing, and the broad flat tooth for grinding offer the two extremes in form; and in passing from the one to the other, we may, by examining the teeth of various animals, observe minute gradations in the change from the vertical to the lateral development, or *vice versa.*

But in all instances we find the jaw beautifully adapted for the most efficient use of the peculiar teeth with which it is armed. So that, from a view of the teeth, we may with certainty predict what would be the form of articulation of the jaw to which they belong, and on the other hand, a view of the articulation will furnish us with means of judging what would be the form of teeth with which the maxilla would be furnished.

The typical form of a tooth, as developed in the mammalian class, is a modified cone or combination of cones; the apex of the cone being sometimes on the masticating surface, at other times at the end of the root, in teeth that are perfected in a short space of time, as the human teeth, the masticating surface, or the base of the crown, will present the base of the cone, while the fang presents its apex; on the other hand, in cases where the development of the tooth is progressive through the whole life of the animal, the base of the cone will be at the edge situated deep in the jaw where the dental development is going on. In man the base of the cone is in the crown of the tooth, and the apex at the extremity of the root, or on the grinding surface.

In the front teeth we have a simple cone when seen from the front, the cutting edge corresponding to the base, and the end of the root to the apex of the cone; seen from the side they present two cones united by their bases, the point of union being the neck of the tooth. The crown and root of the canine teeth present each a cone uniting at the neck of the tooth.

The molar teeth exhibit in their fangs modified cones, which, uniting laterally at their bases, form the neck of the tooth, while the prominences of the grinding surface are formed of lesser cones. Thus, in examining a developing or a recently developed tooth, prior to any alteration of form from use, the tendency to the conical formation is everywhere found.

The teeth in the human subject are arranged in the jaws in two parabolic curves, the one in the upper jaw being the larger of the two. From the greater size of the curve the teeth of the superior maxilla close in the anterior part of the mouth, external to those of the inferior maxilla, and at the sides of the mouth the external edge of the molars of the upper close over the external edge of those of the under jaw.

The crowns of the teeth are arranged in an even line, no tooth rising higher than its fellows; a condition peculiar to man, and arising from the equal development of each tooth; a second peculiarity is found in the absence of intervals between the anterior and the grinding teeth.

The teeth are developed in pairs, one on either side of the mesial line, and amount in the adult, when perfect in each jaw, to thirty-two; the dental formula being, incisors, $\frac{4}{4}$; canines, $\frac{2}{2}$; bicuspides, $\frac{4}{4}$; molars, $\frac{6}{6}$. In the child, prior to shedding the temporary teeth, the number amounts to twenty only, the dental formula being, incisors, $\frac{4}{4}$; canines, $\frac{2}{2}$; molars, $\frac{4}{4}$: why the temporary and permanent set of teeth are unequal in number will be seen when the development of the teeth and jaws is considered.

For the convenience of description, a tooth is divided into three parts; the crown, which is the exposed portion; the neck, which is continuous with the crown, and is covered by the edge of the gums; and the root, or fang, which is a continuation of the neck, gradually diminishing in size to its extremity, and lodged deep in the jaw.

Three tissues enter into the formation of the human tooth; the enamel which coats with variable thickness the crown of the tooth; dentine, which forms the great bulk of the tooth; and cement, or dental bone, which forms the surface of the fang.

In the centre of each tooth is a cavity partaking generally of the shape of the exterior of the tooth, being very small at its commencement in the root, and gradually increasing in size, till it terminates in the crown. The vessels, nerves and remains of the formative pulp, occupy this cavity, from which it has received the name of the pulp cavity.

The crown of each tooth presents five surfaces. First, a masticating surface; second, an external or labial surface; third, an internal or lingual surface; fourth, an anterior or mesial surface, which in the front teeth is directed towards the mesian line, and in the molars points anteriorly. Fifth, the posterior surface.

Each tooth is larger in its labial surface than in its lingual, arising from the greater size of the curve, described by the outer or labial surface, than that formed by the inner or lingual surface of the teeth.

We now come to the consideration of each kind of tooth, beginning with those situated at the anterior part of the mouth, and proceeding in the order of their position backwards towards the angle of the jaws. The description, however, must be general, for as no two teeth of the same kind are *exactly* similar in size and form, a description, taken from an individual specimen, would, when applied to other individual teeth of the same kind, prove incorrect.

The *incisors* are four in each jaw; two central, two lateral. Of those in the upper jaw the central are the larger. The crown presents a large scarcely convex labial surface, is wedge-shaped, from behind forward, and terminates in a broad cutting edge, similar in shape to a chisel. The root is conical, diminishing in size gradually to the apex, and the tooth when seen from the front presents in outline a tolerably perfect cone, the cutting edge forming the base.

If the tooth be examined from the side, it will present the outline of two imperfect cones, united by their bases, the apex of one cone being at the cutting edge of the tooth, and of the other at the end of the root. The side near the mesial line is longer and less curved than the opposite side of the tooth, and the angle formed by the junction of the mesial side and the cutting edge is less rounded and less obtuse than that formed by the opposite side and cutting edge. The anterior surface of the crown is slightly convex in each direction, and is commonly marked by one or two shallow and irregular grooves running in the length of the tooth. The lingual surface of the crown is slightly concave in each direction, but principally from above downwards; the concavity terminates in a ridge or basal ridge, situated at the commencement of the neck, where the tooth passes into the gum. The enamel terminates on the anterior and posterior surface of the tooth in a curved line, the convexity of the curve being directed towards the fang, while in the sides of the tooth it terminates in a curved line with the convexity directed towards the crown. In point of size the central are larger than their neighboring teeth, the lateral incisors, the average length of the former being about one inch. This remark applies to the incisors of the upper jaw only, for in the lower row the lateral are the larger teeth.

*Central incisors, lower jaw.*—Though possessing a general resemblance these are much smaller than those of the upper jaw, being only half as wide as the cutting edge. The diameter of the antero-posterior surface is, however, almost equal. The lateral surfaces rapidly converge to the neck, which is succeeded by a much compressed and slightly grooved root.

The enamel terminates in a curved line on the labial and lingual surfaces, the convexity of the curves being directed towards the root of the tooth: on the lateral surface the terminal curves of the enamel are reversed. Average length eight-tenths of an inch.

*Lateral incisors, upper jaw.*—Are less in lateral diameter than the centrals, frequently to the amount of one-third, the depth of crown and of enamel being slightly less; the mesial surface is more or less concave, while the surface opposed to the canine is slightly convex. The anterior surface is generally convex, and broader at the cutting edge than at the neck of the tooth. The posterior surface presents an inclined plane, directed from behind forwards, whereby the cutting edge is formed, while the neck of the tooth is left thick and strong. The fang is laterally compressed and somewhat shorter than that of the central; the posterior basal ridge is generally absent, though sometimes strongly marked.

*Lateral incisors, lower jaw.*—Are slightly larger both laterally and antero-posteriorly than the central incisors; the depth of the crown in the two teeth being nearly equal, while the fang of the former is slightly the longer.

*Canines, or cuspidati, upper jaw*, placed between the lateral incisors and the first bicuspides are the largest of the single-fanged teeth, and have a more deeply implanted fang and a greater antero-posterior diameter than either of the incisors. The crown terminates in an obtuse point, from whence it gradually increases in lateral dimensions, attaining its greatest diameter at a spot equi-distant from the point and neck, towards which it gradually diminishes in size. The external surface is convex, with a slight ridge, commencing insensibly at the neck, and becoming stronger as it approaches the point, bounded on either side by a depression; a similar ridge also exists upon the posterior surface of the tooth, which, like the central incisors, presents an inclined plane, terminating in a slight basal ridge. Of the two sides, the one next the bicuspid is the more convex; the fang is longer than that of any other tooth; it is compressed laterally, and is slightly grooved at its sides: the length often amounts to one inch and one-sixth. The existence of a groove in the sides shows a slight tendency to the formation of two fangs, and may be regarded as the first step towards the formation of roots. I have in my possession a canine having two fangs.

*Canines of, lower jaw,* differ from those of the upper jaw principally in terminating in a broad point, and in being less convex anteriorly; in having more perpendicular coronal sides, and a fang slightly shorter and more deeply grooved in the sides: the perpendicular ridge in the internal or labial surface is also very slight or altogether absent. The average length is one and one-eighth of an inch.

*Bicuspides, or premolars, upper jaw,* form the next class; and in them we find the most simple form of molar tooth. Of these there are four in each jaw—two on either side; and, from their position, called the first and second bicuspides.

The crown is composed of two cones united laterally, from which proceed a laterally compressed fang, common to each. The labial surface is convex, and, like that of the canine, terminates in an obtuse cone. The lingual, or internal surface, is also convex, and, unlike either of the foregoing teeth, placed vertical to its alveolus. Of the two tubercles or cusps, of the masticating surface, the external is the larger, especially in the anterior bicuspid. The crowns of these teeth are at least one-third shorter than those of the previously described teeth. The fangs are either two in number, corresponding to the two cusps; or if there are not two, the one is very deeply grooved and perforated by two cavities uniting to form one in the crown, or if there be a single cavity, it resembles in section a line dilated at its extremities. In some few instances the single fang is deeply grooved externally as well as on its side, and at the apex divides into three points, thus showing a strong tendency to the formation of three fangs similar in position to those of the molar teeth of the upper jaw.

*The bicuspides, lower jaw*, have some points of dissimilarity when compared with those of the upper jaw. They are generally more rounded, and the labial surface is more convex. The two tubercles of the masticating surface are less distant from each other, the grooves separating them being imperfect, and not carried fully across the tooth. The fang also is more rounded, and seldom or ever double. Indeed, these teeth may be considered as intermediate between the canines and bicuspides of the upper jaw. The first and second are much alike, the second being rather the larger, and having a more developed inner cusp. In the first, the two cusps are connected by a low ridge passing from the one to the other across the centre of the crown.

*Molars, upper jaw.*—There are three pairs of molars in each jaw, upper and lower, named in the order of their respective places, as regards the anterior part of the mouth, first, second, and third. The first and second pair being generally much alike, may be described together. The masticating surface is armed with four cusps, two external or labial anterior and posterior, and two internal or lingual anterior and posterior. Of these the anterior internal is much the largest, and is connected to the posterior external cusp by a low ridge, separating, by its oblique course, the anterior external, and the posterior internal, from each other. The grooves separating the cusps at their bases extend over the external and lingual surface of the teeth, but are lost before they reach those surfaces of the tooth which adjoin the neighboring teeth. The cusps are, however, subject to variety in number, especially in the second molar; thus sometimes several lesser ones appear, while at others the two internal or lingual cusps are united in a large one. The crown of the tooth, disregarding its tubercles, is a four-sided figure, with parallel sides, united with two obtuse and two acute rounded angles; two sides standing parallel with the line of the jaw, while the other two are directed inwards and backwards towards the pharynx on the opposite side. The roots are three in number, two external and one internal. Of the two external fangs the anterior is the larger, and stands a little more outward than the posterior root; they are each compressed laterally, and have their greater diameter from without inward. The third, or palatine fang, is much larger than either of the others; it is broad and thick at its base, and, if there be two inner coronal cusps, grooved as though formed of two conical roots; it is directed obliquely upwards and inwards towards the palates. In some few instances there are four distinct fangs;* on the other hand, it is by no means uncommmon to find the palatine and the anterior, but more frequently the palatine and the posterior fangs, united in one, the points of union being marked by longitudinal grooves. The enamel terminates gradually in an even line round the neck of the tooth.

*Molars, under jaw.*—Of these the first molar is the largest; the grinding surface of this tooth is divided by an irregular cross-shaped fissure into five tubercles, two anterior and two posterior; between the two latter the fifth, a wedge-shaped cusp is situated.

When the tubercles have been worn down by use, the grinding surface offers a tolerably square surface, surrounded by a ring of enamel. The external surface of the crown is usually marked by a vertical groove, which often passes half way down the crown, and ends in a small hole, in which disease often shows itself.

The roots are two, one anterior and one posterior. They are strong, much compressed, and grooved from behind forwards, generally diverge and turn a little backwards, sometimes their backward course is very considerable. I have seen one or two instances in which, instead of two grooved fangs, four distinct fangs have been presented. In the second molar we seldom see the fifth cusp, neither is the tooth so large as the first. The roots are not unfrequently united in one conical mass; in other respects the second resembles the first molar.

*Third molar, upper jaw,* though generally resembling the first and second, is, in most instances, more irregular in form, and less in size. The fangs are frequently compressed into a conical mass, sometimes turning in one direction, sometimes in another. Occasionally the three fangs are separate and well developed but it is not uncommon to find them more numerous,

* Bell.

small, and irregularly bent. The crown is commonly larger from side to side than from before backwards.

*Third molar, under jaw.*—This is a larger tooth than its fellow in the upper row. The crown usually bears four cusps, and resembles the adjoining molars, except that it has a more rounded appearance. The roots are two in number, generally directed backward, and very commonly connate, the lines of union being marked by longitudinal grooves; a similar groove is also seen in the other sides of the root, thus giving evidence of the tendency to the development of four fangs corresponding with the four cusps.

---

### LECTURE II.

RELATIONS OF UPPER TO THE UNDER ROW OF TEETH IN CLOSING THE MOUTH—THE ALVEOLI—NERVES OF THE MOUTH—BLOOD-VESSELS OF THE TEETH—THE TEMPORARY TEETH—THE ARTICULATION OF THE JAWS—MASTICATION—STRUCTURE OF THE TEETH—DENTINE.

GENTLEMEN:—Having in the last lecture examined separately each class of tooth, it will be interesting to review their progressive increase of size and of use, as we proceed from the anterior to the posterior part of the dental arch. The most simple form of tooth is in shape an elongated cone, fixed at its base, and adapted for seizing only. Such a tooth is not found in the human subject, but the next form in the order of complexity of use we possess, namely, a tooth in which a broad cutting edge is substituted for a point. Such are the incisors, single fanged teeth, with cutting edges, those of the upper jaw closing over those of the under jaw, like the blades of scissors, and in such a manner as to keep by use the edge of each tooth tolerably sharp. From the incisors we come to the canines, which teeth, terminating in an obtuse point, and having like the incisors an inclined lingual surface, are adapted from their form and the strong deeply-planted root, for dividing or lacerating tough substances. In these teeth are the first indications of the development of two distinct fangs; thus exhibiting in use and form, a third step towards the more complex teeth. Passing from the canines, the bicuspides present themselves. In these all the developments of the more complex form are observable. The crowns are tuberculated, and the root of the tooth, if not divided in two, is deeply grooved; and in a few examples the bicuspides of the upper jaw have three fangs, similarly arranged to those of the neighboring molars. In use, the bicuspides are equal in the degree of their relative size to the molars which join them. The molars are in form the most complex of the human teeth, having several divergent roots and a tuberculated masticating surface—a conformation admirably fitted for triturating the food divided by the anterior teeth.

*Relations of the Upper to the Under Row in Closing the Mouth.*

The upper incisors and canines, when the mouth is closed, form the larger arch in which they are arranged, shut over, and in front of the lower teeth, concealing the upper third of their crowns, while the external tubercles of the bicuspides and molars of the lower jaw are received into the depressions between the external and internal tubercles of the similar teeth in the upper jaw, thus allowing the external tubercles of the upper teeth to close externally to the outer tubercles of the lower row. From this arrangement of the tubercles, we are enabled in mastication to use the whole surface of the crowns of the opposing teeth; the act of mastication being performed by bringing the external tubercles of the under molars opposite those of the upper row, when, by the lateral motion of the under jaw inwards, the external tubercles pass down the inclinations of the external and up the inclinations of the internal tubercles of the upper jaw teeth, reducing in the action any interposed substance. It will also be observed that, from the difference of size laterally in the incisors of the two jaws, the central incisors of the upper extend over the centrals and half the laterals of the under row, and that the superior laterals lie over the remaining half of the inferior laterals and the anterior half of the canines of the lower jaw. The canines close over the halves of the first bicuspides and canines, while the first bicuspides impinge on the half of the first and of the second bicuspides of the lower row. The second bicuspides close upon the anterior third of the opposing first molar, and the posterior half of the second bicuspid. The first molars oppose the posterior two-thirds of the anterior, and one-third of the second molars of the lower jaw, while the second upper molars close upon the unoccupied posterior third of the second and the anterior third of the wisdom teeth. The wisdom tooth of the upper being smaller in size than that of the lower jaw, is perfectly opposed by that portion of the latter left unoccupied by the anterior tooth. By this admirable arrangement, no two teeth oppose each other only, but each tooth in closing the jaws impinges upon two, so that should a tooth be lost, or even two contiguous teeth, still the corresponding teeth of the opposite jaw are to some extent opposed, and thus remain useful. For when a tooth is wholly unopposed, a process is set up in the jaw by which the useless organ is gradually ejected. The direction of the teeth in the upper is vertically downwards and slightly forwards, while those of the lower jaw are placed vertically, the molars bending slightly inwards.

### *The Alveoli.*

Having become acquainted with the external form of the various classes of teeth, we shall now consider the alveoli, or sockets in which they are implanted. The alveolar processes bound the inferior border of the upper jaw and the superior edge of the under jaw, and form curves more or less elliptical. They consist of an external and an internal plate, connected by transverse septa, the internal being the stronger of the two. The spaces between the septa receive the roots of the teeth, to which they are accurately moulded. The latter having been described, it is needless to repeat the description of their reverses. The septa are composed of less dense bone than that forming the external or internal alveolar plates, and are somewhat longer. At the bottom of each alveolus is found one or more foramina, through which the vessels and nerves pass in their way to the tooth. The external plates of the alveolar processes are in the anterior part of the jaws, irregularly fluted, the depressions corresponding to the septa. In the upper row the external convex surface of the roots of the teeth are sometimes near the apex without alveolar covering, but the neck is always enclosed.

The free edges of both the external and internal alveolar plates have a festooned margin, the concavities being opposite to the alveoli, and directed towards the crowns of the teeth. The walls of the alveoli are perforated by innumerable small holes for the transmission of vessels to the periosteum, by which the roots of the teeth are connected to their close fitting sockets.

The surfaces of the alveoli are every where closely invested with periosteum, which on the free surface is clothed with mucous membrane continuous with that of the mouth, and thereby forming the gums, which, having invested the external surfaces of the alveoli, attach themselves to the necks of the teeth, and there terminate in a free edge which lies over the neck of the tooth and the terminal line of the enamel. The structure of periosteum and mucous membrane will be described by your lecturers on anatomy.

The kind of articulation by which the teeth are fixed is termed by anatomists *gomphosis*. The teeth are retained in their position first by the close adaptation of the alveoli to the fang, and secondly by the strong adhesion of the periosteum to the walls of the alveolar cavity and to the surface of the root. Where the fangs diverge, or are crooked, the teeth are held firmly by the corresponding shape of the alveoli, and offer strong resistance to the force employed for their removal. We shall, however, recur to this part of the subject when treating of the extraction of teeth. But it must not be supposed that the teeth in their healthy state admit of positively no motion. By taking a tooth by the thumb and finger it may be made to move, though slightly yet perceptibly, from side to side, arising from the compressibility and elasticity of the alveolar periosteum. This elastic tissue no doubt performs a double function; first, in contributing to retain the teeth in their place, and maintaining their vitality, and secondly, in diminishing the shock communicated to the jaws by the sudden closure of the teeth.

### *Nerves of the Teeth.*

The teeth of each jaw receive their nerves from sensitive branches of the fifth pair. The molars of the upper jaw are supplied by the posterior dental, a branch from the superior maxillary nerve before its entrance into the orbit, which branch, dividing into twigs, passes into the substance of the bone by small foramina at the posterior part of the tubercle. The superior bicuspides and canines, and incisors, are supplied by two branches, the middle and anterior dental, given off during the passage of the nerves through the infra-orbital canal. The third division of the fifth nerve furnishes the inferior maxillary by which the teeth of the lower jaw are supplied. The inferior dental nerve enters the jaw by the posterior dental foramen, and lies in its course immediately under the roots of the teeth, to each of which it sends off a small branch, which entering the root through a small foramen, is distributed to the pulp. The three superior dental from the infra-orbital nerve anastomose by their primary branches, from which, and from a plexus formed by the secondary branches in the lining membrane of the antrum, two sets of nerves, directed one set (rami dentales) to the teeth, the other (rami gingivales) to the osseous tissue and gums.* The infra-orbital and the inferior maxillary nerves, both before and after the origin of the dental branches, give off nerves to be distributed to the parts about the face, with which the dental branches anastomose; thus establishing links of connection by which sensation may be transmitted from one part to another, throughout the whole course of the nerve. By tracing these intercommunications of the nerves, we are able to understand the various sympathetic pains so frequently manifested in odontalgia, and in neuralgia and rheumatic pains, between the teeth and the neighboring parts.

### *Blood-Vessels of the Teeth.*

The teeth are dependent upon the *internal maxillary* branch of the external carotid for their connection with the arterial system.

* Swan on the Nerves.

The molars of the upper jaw receive their vessels from the superior maxillary, which sends in small branches at the posterior part of the maxilla. The anterior teeth of the upper row receive vessels given off from the infra-orbital artery.

The inferior maxillary artery passing downwards between the pterygoid muscles, enters the posterior maxillary foramen, and, accompanying the dental nerve, gives branches to the roots of the teeth which enter the dental foramina with the nerves, and are distributed to the pulp. The artery ultimately emerges with the nerve at the mesial foramen, and is distributed to the chin and lips, where it anastomoses with branches of the facial.

The dental arteries, in addition to the branches given to the teeth, contribute to supply the alveoli and neighboring tissues.

The *milk teeth* are altogether smaller than their successors, not only in size, but also in number; the dental formula being, incisors, $\frac{4}{4}$; canines, $\frac{2}{2}$; molars, $\frac{4}{4}$. In the child there are no bicuspides, the space destined for them being, at this period, occupied by the temporary molars. The incisors and canines, in form, much resemble the similar teeth of the adult. One point of difference has, however, been observed by Mr. Saunders, in his lectures,* namely, that the enamel terminates on the neck of the tooth by a thick edge, as compared with the terminal line of the enamel of the permanent teeth. This observation applies equally to all the temporary teeth. The milk canines in the upper jaw, except in size, are like their successors, but those of the under jaw are more curved than the permanent teeth, and have their lateral surfaces curved, as in the canines of the upper jaw. The deciduous molars are altogether dissimilar to their successors, the bicuspids, but in the number of their roots, and to some extent in the form of their crown, resemble the permanent molars. The *anterior molars* have crowns flattened laterally, both in the lower and in the upper row. In the upper they are furnished with three cusps, two external and one internal; the latter being the larger. In the under teeth, the crown is indented by a crucial fissure, situated at the base of five cusps,

* Lectures on Diseases and Operations of the Teeth, by Edwin Saunders, Esq.—The Forceps, 1844.

the two anterior being the larger. These, the first temporary molars, resemble, therefore, the second, rather than the first permanent teeth of the same name. *The second milk molar* resembles, in the shape of its crown and in the number and disposition of its tubercles, the first permanent molar. Thus, in the upper jaw, the anterior internal and posterior external are united by an oblique ridge, while, in the lower teeth, we have a crucial fissure at the base of four cusps, with a fifth cusp wedged in between the two posterior cusps. In addition to the peculiarities already enumerated, we find that the roots diverge from the neck of the molars at a greater angle than do the fangs of the adult teeth. Although the temporary are less in size in each direction than the permanent molars, yet they are greater in their antero-posterior diameter than their successors, the bicuspids; so that the latter teeth, as they disappear, leave space anteriorly from the permanent incisors and canines, which, being larger, occupy more space than that vacated by their predecessors.

The portion of the jaw occupied by the milk teeth, corresponds, in size and shape, to the anterior parts of the adult maxilla occupied by the incisors, canines and bicuspids; and the line described by the outside or labial surface of the teeth is in each a semicircle. The permanent molars are developed posterior to the milk molars, and are not, like the anterior teeth, preceded by temporary teeth. This, however, will be explained when speaking of the development of the teeth.

Before leaving the descriptive anatomy of the teeth and alveoli, it will be well to consider the form of articulation of the jaw, by which the organs of mastication are made to operate upon the substances submitted to their action. As we have the extreme of form in teeth adapted, the one for simply piercing, the other grinding, so we have corresponding extremes in form of maxillary articulation; the one admitting of motion in the vertical plane only, in which articulation the jaw can be opened and closed only, the other allowing motion not only in the vertical, but also, to some considerable extent, in the horizontal plane, in which form of articulation, the teeth being closed upon the food, are moved laterally; in this action, the surface of the opposing teeth rubbing forcibly upon each other, reduce to small particles the interposed food. In the carnivora, where lateral motion is not required, the condyle of the inferior maxilla is narrow from behind forward, broad laterally, and is closely embraced by the glenoid cavity, which is not larger than is required for the reception of the condyle; but in many of the herbivorous animals the condyle is broader, and is received in a large flat glenoid cavity, in which it can move forward and backward, as well as upon its axis.

In producing the lateral motion, one condyle is moved towards the posterior margin, and the other is advanced towards the anterior margin of the glenoid cavity. In the human subject is found a form of articulation intermediate between the two extremes, and is thus in strict harmony with the teeth which are of intermediate development between the carnivora and herbivora. In the infant, prior to the eruption of the teeth, the maxillary articulation admits of scarcely any lateral motion, which inability arises from the glenoid cavity being but slightly larger than the received condyle.

In old age, after the loss of the teeth, the lateral motion, from want of use, is much diminished, if not altogether lost. After the loss of the teeth, and the consequent absorption of the alveolar processes, the distance between the chin and nose on closing the jaw is diminished, and, as ordinarily computed, to the extent of an inch and a half. In this state, when the mouth is opened, the under jaw is seldom depressed lower than to the point which it occupied in the closed state, when armed with teeth. The condyles are, therefore, seldom advanced to the anterior margin of the articular cavity, and as parts out of use cease to be capable of use, so the absence of necessity for use is followed by inability. Thus the loss of the teeth operates as a cause in diminishing the power of motion in the aged jaw, first, in destroying the necessity for lateral motion, and secondly, in diminishing the amount of the vertical action of the jaw.

### *The Structure of the Teeth.*

Of the various tissues with which the human frame is constructed, none are more beautiful or more instructive to the inquiring physiologist than those forming the teeth, and at the same time none are more easy of demonstration, if the microscope be used in the investigation. As I before observed, the dentinal tissues are three—namely, dentine, or tooth-substance; enamel, and cement, or more properly, tooth-bone.

The enamel invests the more prominent parts of the crown, from which points it gradually diminishes in thickness, till it terminates in a line on the neck of the tooth. The cement, or dental bone, is thickest at and near the end of the root, and gradually becomes thinner as it advances towards the crown of the tooth. In a tooth that has been used for some little time, the cement terminates where the enamel commences, but there is reason to believe that a thin layer is continued over the enamel. Of these tissues, dentine, as forming the great bulk of the tooth, and thereby becoming the most important, will first demand our attention. The pulp cavity occupies the centre of the dentine, and on its surface are superimposed the enamel and the tooth bone, the former investing the crown and the latter the surface of the fang. These two tissues form a layer of variable thickness in different parts of the teeth. This layer, however, is soon worn off when the tooth comes into use. If the enamel and cement be removed from a tooth, and the dentine alone allowed to remain, still the tooth retains much of its original shape, losing most at the two extremities, while, in point of size, the loss sustained is comparatively slight. Thus showing the dentine to constitute by far the greater portion of the tooth.

FIG. 1.

FIG. 1.—Longitudinal section of a canine tooth, showing the three dental tissues. *a.* The dentine, or ivory. *b.* The enamel. *c.* The cementum, or dental bone.

The dentine is made up of two distinct parts: first, dentinal tubes; secondly, in the tubular tissue. The tubes have distinct parietes, equal in thickness to their calibre. In some instances they appear to contain a minute granular matter; but in many, perhaps in the majority of cases, they are perfectly free from solid contents. If a vertical section, passing through the pulp cavity, be taken for examination, the dentinal tubes may be traced from their commencement on the surface of the pulp cavity to their termination at the junction of the cement, and the dentine or the enamel and the latter, or they may be seen passing into these external structures. The tubuli commence at right angles with the surface of the pulp cavity, and proceed outwards towards the surface of the tooth, giving out in their way numerous small branches, which, meeting with other similar branches from neighboring tubes, anastomose with them, or, meeting with simple cells in the intertubular tissue, there terminate. Towards the surface of the dentine it is not uncommon to see a tube reverse its course, and, by joining another, form a loop. The tubes all commence in the pulp cavity, and pass outwards towards the surface of the dentine. Their course, as regards each other, is divergent, so that the proportion of the intertubular tissue increases relatively as the distance from the pulp cavity is greater or less. This preponderance of the intertubular over the tubular tissue near the periphery of the tooth is, however, in a considerable degree lessened by the more frequent branching of the tubes, and by the occurrence of cells near the surface of the dentine. If a single tube be traced through its whole extent, it will be found to make two undulations, and in addition to these, which are called the primary curves, a number of smaller undulations.

FIG. 2.

FIG. 2.—Dentinal tubes as seen by a low power, showing the primary curves, *a*, the secondary curves, *b*, and the branchings with the anastomosis of the branches. *c*. A tube assuming the form of a bone corpuscle.

In examining this structure, a thin section may be taken from the fang, and, with the aid of the microscope, viewed by transmitted light A tube will then appear as a very definite dark line pursuing its tortuous but definite course towards the surface, giving out numerous minute branches on its way, and at last dividing into two terminal branches, which end either by passing into a cell of the intertubular tissues, or by anastomosing with a collateral tube, or by passing into the cement. If the section be taken of the dentine and enamel, then the tubes will be seen to give out comparatively few branches till they come near the latter, when they divide and anastomose freely, and some few terminal branches may be traced entering the enamel. It is by no means uncommon for a tube in its course to suddenly dilate and give out branches from the dilatation; then again contract, and pursue its original course.

FIG. 3.

FIG. 3.—A longitudinal section of dentine, showing a dentinal tube dilated, and sending off anastomosing branches to the neighboring tubes.

In such a case the dilatation forms a cell in every way similar to the bone cell or corpuscle, of which some account will be given when we trace the structural relations of dentine and bone. When seen by transmitted light, that is, when the object examined is placed between the light and the eye, the tubes appear as dark lines, unless a very high magnifying power be used, or unless the tubes are very large, such as are found in the teeth of some fish; take, for example, the wolf fish; there the tubes are marked by two parallel dark lines, with a narrow line of light interposed. Instead of this appearance, a sub-granular matter, in some cases, seems to occupy the area of the tubes.

FIG. 4.

FIG. 4.—Longitudinal section of dentine highly magnified. *a*. The dentinal tubes. *b*. The intertubular tissue, in which is shown the granularity.

FIG. 5.

FIG. 5.—Transverse section of dentine. *a.* The tubes, with their parieties and area seen. *b.* The intertubular tissue.

The dark line so constantly seen with a low power is, I think, no proof that solid matter is contained in the tube, for the appearance described may be exactly imitated by introducing minute globules of air into a fluid, and then placing it on the field of the microscope, when, by transmitted light, each air globule will be bordered by a dark line, while the centre is light. The dark line, in this case, is dependent for its origin upon the interference of light produced upon the ray of light—by the curvilinear form of the globule, which refracts the light at such an angle that it precludes the possibility of the rays passing through the lenses of the instrument. If the dentinal tubes be examined by reflected light; that is, if the section be placed upon a dark ground, and the light be made to fall upon it instead of passing through, as in the former experiment, then the tubes will be seen as opaque white lines placed in a transparent medium. This appearance would at first sight seem to indicate that the tubes are filled with solid granular contents; however, in this as in the former instance, the appearance can be imitated without the presence of granular matter. If a transparent substance, as glass, be taken and reduced to powder, and the powdered mass be examined by reflected light, it will appear as an opaque white substance, studded with transparent particles. In this instance the opacity is clearly due to the refraction from the minute particles being at such angles that the rays of light pass external to the field of the glass. But the dentinal tubes may be rendered perfectly transparent if they be filled with transparent substances of greater density than air. This may be effected by immersing sections in Canada balsam or spirits of turpentine. In each of the foregoing methods of demonstrating the dentine tubes, the areas only are seen; but if a section be taken external to, but parallel with, the length of the pulp cavity (in which cases the tubes will be cut transversely,) then the parieties of the tubes will be seen with the intertubular tissue, in which they are impacted.

In the year 1837, I was engaged in examining the structure of teeth, and then came to the conclusion that the dentinal tubes contained an amorphous salt of lime. My opinion was grounded on the following experiment :—After preparing a thin section of human tooth, I placed it in the field of the microscope, and then added a little diluted muriatic acid. No sooner was the acid in contact with the section than evidence of chemical action was rendered visible by the appearance of bubbles of gas, and these emanating not only from the external surfaces of the section, but also from the interior of the tubes, from which bubbles of gas were seen issuing in quick succession. When the action ceased, the tubes no longer presented the appearance of opaque dark lines, but were indistinctly seen filled with transparent fluid. At the time, much struck with the result of the experiment, I was led to the opinion, that the gas generated in the tubes was produced by the decomposition of their solid contents, which I supposed to be carbonate of lime ; however, upon more extended observation, I was induced, from the examination of teeth, in which the tubes are large, and also from the examination of the tubuli of the human tooth with high power, to modify my first opinion, and was compelled to adopt the views advanced in the former part of the lecture, and to regard the evolution of gas from the tubuli as evidence of the facility with which fluids are admitted into their interior, and to consider that the source of the gas existed in the decomposition of the parietes of the tubes rather than of their contents.

The point of greatest diameter of the dentinal tubes is at their commencement on the walls of the pulp cavity, though in their course previous to the division of the trunk into two terminal branches they suffer but little loss in calibre.

In tracing this structure in the teeth of various animals we find every form of branching; sometimes the branches are few, in others extremely numerous ; in one instance given out from one side of the tube only, in another from each side ; but whatever the modification in the number or form of the branching given out, the primary tube always commences by an open extremity on the walls of the pulp cavity, or upon the walls of a canal for a blood-vessel ; and the direction taken by the tubes is invariably towards the periphery of the tooth, always anastomosing in their way by the numerous branches.

In the temporary, and not unfrequently in the permanent teeth, the tubes instead of presenting an uninterrupted line present on their surface numerous indentations just as though they were composed of a series of hollow beads, which were united and made to communicate with each other. This appearance has led Mr. Nasmyth to conclude that the teeth instead of being composed of tubes are made up of baccated fibres,* of which he gives a drawing, separated from the intertubular tissue. This appearance, however, admits of another explanation, which will be given after the development of the dentine has been explained.

---

### LECTURE III.

GENTLEMEN :—At our last lecture we were engaged in considering the dentinal tubes; we shall now give our attention to the uniting medium of the tubes.

The second part composing the dentine is the intertubular tissue, which occupies the spaces between the tubuli, everywhere surrounding and investing them, and thereby contributing greatly in rendering the whole dentine a solid dense mass, the area of the tubes and cells being the only hollow portion. It would be difficult to estimate beyond mere guess the relative amount of this structure in a tooth as distinguished from the parietes of the tubes, since it is only seen in a transverse section of the latter, where the area of the tube is in appearance about equal to the diameter of its parietes, and the intervening space between the two tubes about equal to the diameter of a tube. This (intertubular) tissue, in a favorable specimen, I have seen composed of very minute granules united to each other on all sides, thus forming a solid mass, of which in character of formation, oolite would give a coarse illustration. The granularity is best seen near the external surfaces of perfect dentine, or in the tissue when devel-

* Memoir on the Development of the Teeth, by Alex. Nasmyth. 1842.

oping. In the intertubular tissue hemispherical or elliptical cells are found, especially near the surface of the dentine of the fang, where they form a layer joining the cement. This, in a paper read before the Royal Society, I described as the granular layer: on the coronal surface of the dentine they are not numerous. With these cells the dentinal tubes communicate, as do others coming from the cemental cells. This point will, however, be further examined when the relations of the dentinal tissues are considered.

We have seen that the parietes of the pulp cavity, under a high magnifying power, present a surface perforated by innumerable minute pores, each pore being surrounded by a circular line, and a tissue intervening between the circular lines of the various pores. The coronal surface, on the contrary, presents a series of minute depressions of hexagonal form. In addition to these, this surface is marked by larger undulations. Into these minute depressions the ends of the fibres of the enamel are received. At present the dentinal tubes and cells, and the pulp cavity, have alone been described as existing in the dentine; but in addition to these, we have, in many instances, canals for vessels traversing the tissue, just as we have the Haversian canals perforating bone. In the tooth of man these vascular canals are never numerous, and occur only in a few teeth. (Fig. 6.) I have seen one or two specimens only in which they traverse the dentine.

FIG. 6.

**FIG. 6.—Longitudinal section of the fang of a human tooth, in which is shown vascular canals traversing the dentine.** ***a*, The dentine; *b*, the cementum; *c*, vascular canals.**

In some animals, however, teeth are found in which the dentine is, in all cases, vascular; the teeth of the walrus offer an example, as do others of the kangaroo and the rabbit. In those instances of vascular canals traversing the dentine which have come under my observation, their direction has been from the pulp cavity towards the surface of the root of the teeth. It must be borne in mind that at present we have been speaking of the dentine alone, and not of the cement, which is generally vascular.

In the teeth of old persons, or in teeth that have been much worn, the pulp cavity becomes much diminished in size, or wholly obliterated by what may be called a secondary development of dentine. The pulp, in such cases, is formed into dentine, the new uniting or not with the previously formed tissue. Such dentine is usually traversed by vascular canals, around each of which the characteristic branching tubuli are arranged radially, as those of the body of the tooth were arranged around the pulp cavity.

Chemically, the dentine is, according to Bibra, composed of the following organic and inorganic substances.

*Incisors of Adult Man.—Dentine.*

| | |
|---|---|
| Organic substances . . . . . . . . | 2828.78 |
| Inorganic substances . . . . . . . | 71.30 |
| | 100.00 |

*Molars of Man.*

| | |
|---|---|
| Phosphate of lime, with a trace of fluate of lime . | 66.72 |
| Carbonate of lime . . . . . . . . . | 3.36 |
| Phosphate of magnesia . . . . . . | 1.08 |
| Salts . . . . . . . . . . . . . | 0.83 |
| Chondrine . . . . . . . . . . | 27.61 |
| Fat . . . . . . . . . . . . . | 0.40 |
| | 100.00 |

Like bone, the dentine is colored by feeding the animal on madder. After mixing a large proportion of madder in the food of a young pig about six weeks old, for a period of three weeks, the animal was destroyed, and the teeth, on examination, were found deeply colored. A section showed that the dentine of the pulp cavity, as well as the cement of the surface of the fang, had been equally affected with the osseous tissue of the skeleton. But I could not discover that the color extended further in the tubuli than in the outer tubular tissue. Neither did the opaque line which marks the interior of the tubes, as seen by reflected light, seem colored.

*Structure of the enamel.*

The enamel, the hardest of the dental structures, is composed of dense semi-transparent fibres, placed side by side, and closely united. Their form is an approximation to a six-sided prism, and their size tolerably uniform, being about the $\frac{1}{4600}$th of an inch in diameter. The direction taken by the enamel fibre is for the most part vertical to the surface of the dentine upon which it rests; those, therefore, which proceed from the flat surface of the crown, will rise vertically, while those from the lateral surface of the tooth will be horizontal. Where the coronal surface of the dentine is concave, the enamel fibres of the opposite sides of the concavity form with each other angles, and meet at their external ends. This juncture is frequently imperfect, and leaves a fissure, under which the dentine, being less protected from external influence than on the other parts of the crown of the tooth, is more frequently attacked by disease. The fissures on the crown of the molars are often subject to this defect of development. The ends of the enamel fibres are received into the shallow hexagonal depressions of the coronal surface of the dentine, from whence, in their course, they frequently describe curves. The direction taken by neighboring fibres is not, however, at all times perfectly parallel; indeed, they often diverge, or cross each other at considerable angles. The curves also seem less regular than those formed by the dentinal tubes. Near the surface of the dentine, small linear interspaces not unfrequently exist between the enamel fibres. With these the terminal branches of the dentinal tubuli often communicate. When the interspaces exist in a great number, and extend to the surface of the enamel, they produce a pearly white appearance, and render the texture comparatively friable. This condition is indicative of imperfect development, and is followed by early decay in the affected teeth. The enamel fibre is not in all cases solid, but has running through the whole or part of its length an extremely minute cavity. This is best seen in newly-developed enamel, but a trace of the canal may be seen in that of adult teeth. Interposed between the individual fibres of the tissue under consideration are the remains of the membrane in which the development has taken place, and which, when hardened by the reception of earthy matter, serves to connect the fibres. This tissue, however, is not traceable except in imperfectly developed enamel, unless by the aid of acids. Transverse markings are sometimes observable upon the surface of the enamel fibres, but they are often indistinct, and their nature, though little understood, is probably connected with the development of this substance. An appearance resembling transverse lines is frequently seen in a specimen, which is traceable to the obliquity of the section, by which lines at equal distances are formed by the cut edges of the enamel fibres. The enamel in the incisors yields to chemical analysis 3.59 of organic, and 96.41 of inorganic matter.

Enamel.—*Molars of Adult Teeth.*

| | |
|---|---|
| Phosphate of lime, with a trace of fluate of lime . | 89.82 |
| Carbonate of lime . . . . . . . . | 4.37 |
| Phosphate of magnesia . . . . . . . | 1.34 |
| Salts . . . . . . . . . . . . . | 0.88 |
| Chondrine . . . . . . . . . . | 3.39 |
| Fat . . . . . . . . . . . . . | 0.20 |
| | 100.00 |

*Tooth.—Bone or Cementum.*

Where the enamel ceases to encase the dentine, the cement commences in a layer, gradually increasing in thickness to its termination at the apex of the root; though, as before stated, a very thin layer is continued over the crown of the tooth investing the enamel. Yet the amount is so small (and even this disappears so quickly after the tooth comes into use) that its existence may be regarded rather as rudimentary than as holding any importance in the human teeth.* In many animals the cement is continued over the enamel in a thick layer, and acts an important part in uniting into a solid tooth a series of lesser ones, or in filling up spaces between highly-developed tubercles, and then producing a continuous surface. The molar teeth of the elephant afford us a good example. In structure, the tooth-bone, or cement, is similar to osseous tissue, its substance being composed of minute granules closely united. Scattered through the so-formed tissue are cells from which numerous tortuous tubes proceed, the tubes themselves freely anastomosing with each other, and with those formed by neighboring cells, by which arrangement, a net-work of cells and tubes, permeable by fluids, is carried throughout the whole mass. When the

FIG. 7.

FIG. 7.—Section of cementum of the fang near the surface of the tooth, showing the cemental cells with their branching tubes; also, the connected arrangement of the cementum. *a*, Cementum; *b*, a highly magnified view of a cemental cell, and of the granular intercellular tissue.

cement exists in any quantity, it is traversed by canals for blood-vessels. I have several specimens of healthy molar teeth from the human subject, in which these canals exists. This general description of the structure is equally applicable to tooth-bone

* Memoir read before the Medico-Chirurgical Society, by Alexander Nasmyth, Esq., January 22d, 1839.

and osseous tissue. I will now confine my observations to the former. The cement, in encasing the dentine, follows the curved surfaces of the fangs, often uniting in one conical mass two or even three fangs, just as we have seen it connect many lesser teeth or denticles in one large one. The roots of the second and third lower molars, and the dens sapientia of the upper jaw, have not unfrequently three fangs thus connected, (see fig. 8.)

FIG. 8.

FIG. 8.—Transverse section of molar, in which the fangs were connate with an Haversian canal traversing the cementum. *a*, the dentine; *b*, vascular canal of the dentine; *c*, the cementum; *d*, Haversian canal of the cementum; *e*, *e*, pulp cavity.

Where such an union exists, the amount of cement is double, being in fact, the two layers of the two fangs united by what seemed otherwise to have been the external surface of the cementum. In such teeth the cement is commonly pierced by one or more canals for blood-vessels, (shown in fig. 8, *d*.) Upon the neck of the teeth the cement exists, but in a thin layer, and is here traversed by minute tubes only, and these, commencing on the surface, pass horizontally inwards towards the dentine; but further down the root the layer thickens, and then the cement is hollowed by cells with branching tubes, their number being in proportion to the amount of cement. If the layer of cement be further thickened, we find it provided with canals for blood-vessels. In arrangement the tooth-bone presents the appearance of laminæ concentrically placed, the centre of the tooth being their common centre; or should a vascular canal exist it is surrounded by concentric laminæ—in this respect resembling, in its laminated arrangement, osseous tissue. The cells are scattered through the cement with some degree of regularity, generally, though not always, following the course as though placed between the laminæ.

The majority of the radiating tubes of the cells pass, either towards the surface of the tooth, or, when such exists, towards the surface of a canal for a blood-vessel. Many branches also go towards the dentine, and anastomose with the terminal branches of the dentinal tubes, while a few follow the course of the length of the tooth, anastomosing freely with tubes pursuing a like direction. Frequently, however, a cell with its tubuli resembles a tuft of moss, the tubes taking in a mass one direction only, and that towards a surface upon which blood-vessels pass. In size the cells have very little uniformity, varying from the $\frac{1}{1500}$ to the $\frac{1}{2000}$ of an inch. The form is generally oval, sometimes round, and occasionally fusiform. The traversing tubes are large at their commencement, but quickly assume a smaller diameter, which they retain to their termination, Mixed with the cemented cells I have occasionally found tubes which pass across the cement towards the surface of the tooth, and present the peculiarity of having fewer branchings, though equal in size to dentinal tubuli, and no doubt performing a similar function. Occasionally, however, the smaller tubuli of the cells enter them. Of the three dental tissues, the cement is softest and contains the largest amount of animal matter, analysis giving 29.42 of organic matter, and 70.58 of inorganic. In the teeth of lower animals, especially in the Edentata, in which enamel is absent, the cement forms a larger portion of the tooth. The cement when exposed is highly sensible to the touch, unless from any cause it has lost its vitality. When from recedence of the gum a tooth is unprotected, pressure with the nail upon the exposed surface will produce severe pain, which sometimes endures a short time after the removal of the cause.

Having described especially the dental tissues, I will now give you some account of the properties they possess in common, of their relation to each other, and of their reactions to the vascular system.

It will be recollected, that, strictly speaking, all tissues are in themselves extra-vascular, that vessels do not permeate their substance, but pass only between their fibres, laminæ or granules, whatever be the structure of the tissue. Thus, in muscles, capillaries pass in the interstices between the primitive muscular fibres; in bone they pass betwen the laminæ; and in the brain between the tubes and granules. But we regard a tissue to be highly organised, or not, in proporton to the relative frequency or absence of capillaries and vessels in the interstices. Thus we speak of the highly organised tissue of the brain, from the vast number of capillaries which traverse at short intervals its substance; while from their absence we regard the cornea as possessing a less degree of organization.

Taking the relative frequency of vessels in a tissue as an index of the degree of its organization, teeth will be placed near the bottom of the scale, but different grades will be given to their three component tissues.

All that seems necessary for the healthy existence of a tissue is the proximity of a vascular current; more or less close to the individual elements according to the character of the particular tissue in question. But in tissues where frequent interspaces for vascular currents would interfere with the functions, we find in the absence of vessels special arrangement providing for the due nutrition of the part. In no instance are these arrangements more beautiful than in osseous and dental structures; for in each of these their functions require that there should be great power of mechanical resistance. In the tooth we find that the centre is hollowed in the form of an arch, in which lie free from injury the dental vessels and nerves, while the tubes and fibres of the dental substance are placed vertical to the surface of the arch, thus giving to the whole and each tube or fibre the position in which their greater power of resistance exists, and at the same time providing for the nutrition of each part by the permeability of the tubes, which passing from the vascular surface, radiate, and by their branching pass to every part of the tooth, not even excluding the enamel.

To return to the consideration of the relative degrees of organization of the dental tissues. The cement or tooth-bone when collected in any amount is possessed of vessels, as well as with cells and radiating tubes in connection with the vascular surface. To this element of the tooth we must, in accordance with the above plan, give the highest place. The dentine, possessing sometimes, though not constantly, vessels, has in all cases its tubes or capillary pores opening directly upon a vascular surface; this then must be considered as the second, while the enamel itself without vessels is only connected with a vascular surface, by the intervening dentinal tubes, and holds the third or lowest degree of organization of the three dental tissues.

If the relative density of tissues be in proportion to the low degree of vitality, still the dental substances will hold the above arrangement. Again, if the relative sensibility of tissues be regarded as an index of their degree of vitality, still the same places must be accorded to the cement, dentine and enamel.

It is a law of nature, that in passing from one form of organised matter to another, no sudden transition shall be made, but that the individual changes shall be so gradual as to be almost imperceptible.

This law we find beautifully exemplified in the gradual change of structure in passing from the cement to the dentine, and from the latter to the enamel.

The cement and dentine possess so many properties in common, and are often so like each other, that in some specimens it is difficult to determine to which of the two tissues the various parts belong. Thus, in the cementum, we find tubuli terminating in open mouths upon a vascular surface, such as the surface of the root of a tooth, while in the dentine we observe the presence of cells with radiating tubes: indeed, we see the dentinal tubuli themselves taking the form of cells with tubular branchings. In the tooth of the kangaroo the two tissues are fairly mixed. Numerous vascular canals pass from the pulp cavity to the exterior of the tooth, and each canal has scattered round it cemental cells, the radiating tubes of which either pass into the canals or connect themselves with the dentinal tubes.

From the facts which I have laid before you, we are led to infer, that the dentine is but a modification of the cement; that the dentinal tubes are but elongated cemental cells, and that this elongation is necessary to enable the tooth to perform its allotted function.

In tracing the relations existing between the dentine and enamel, we find the change in passing from one structure to another equally gradual.

For illustrations recourse must be had to the teeth of fish, in which the two structures very nearly resemble each other. In man the tubes send branches into the enamel, but the two structures being each highly developed, present points of marked dissimilarity.

From what has already been said of the dental tissues it will be seen that the area of the tubes, of the cemental cells, and of the tubes or interspaces of the enamel, form no part of the tissues themselves, but are in fact spaces in them. These spaces we have seen are of characteristic form in each tissue, and we have considered their relations, &c. &c. It now remains to say a few words on the structural relations of the dental to other tissues. Before going to this point, allow me to state in a few words the ultimate structure of osseous tissue, in order that in comparing the dental structures with each other, we may understand their relations to the tissues to which they are most nearly connected.

Bone is composed of extremely minute granules, closely united to each other, and so disposed as to form laminæ. The laminæ are concentrically arranged, the inner layer forming the parietes of a tube for the transmission of one or more vessels. Between, or in the lines of the laminæ, cells, of an oval or round shape, flattened on their sides, occur, from which proceed numerous minute branchings: tubes which are directed either to a vascular surface, on which to end by an open mouth, or are directed towards other tubes, with which to anastomose, thereby connecting the cells. Thus, in bone, as in the cementum, and in dentine, and indirectly in the enamel, we find a set of capillary tubes, commencing upon a surface, bathed by vascular currents, and passing into the structure, and there establishing a perfect continuous network of tubes, so that a fluid may pass through the whole mass. That this arrangement is subservient to the nutrition of the texture is sufficiently apparent, when we consider its relations to the vascular system, coupled with the fact, that these tubuli must be filled with fluid, even by atmospheric pressure, and that the only source of fluid is the blood.

That the tubes do contain fluid is proved by the following experiments. After removing several teeth, I carefully wiped the external surface; I broke the tooth, and removed the pulp, and made the surface dry. The fragments of dentine and cement (the enamel having been in great part broken off) were then placed in a warm room to dry, and in the course of a few hours lost one part in eight by weight, without having suffered any loss in bulk.

From these considerations it is seen that osseous and dental tissue are in the form and arrangement of their cells and tubes very closely allied, but the relations of the ultimate tissues are yet closer; for dentine and cementum, and probably enamel, are built up, like bone, of more or less spherical granules, the difference in the tissue being in the relative quantity of earthy matter with which the granules are impregnated.

Till lately but little was known upon the structure of the teeth, and that little was overlooked. Luenooke made out the dental structure to be tubular, but his observations were disregarded until* the subject was taken up by Purkinje. Professor Retzius, of Stockholm, about the same time, pursued the minute anatomy of the teeth, and with similar results to Purkinje, which were published in Stockholm in 1836. Late in the year 1837, prior to the appearance of the works either of Purkinje or Retzius in this country, I commenced the investigation of the structure of the teeth, under the impression that little or nothing was known upon the subject. The whole of my time which was not occupied on lectures, or necessary professional study, (I was then a pupil at King's College,) was devoted to the pursuit of dental anatomy, and in a short time I found many points, as I then thought, quite unknown. After I had exhausted the subject, so far as human teeth were concerned, and examined the teeth of all the common animals, I became acquainted with Professor Owen, who, after examining many of my preparations,

* De penitiori dentium humanorum structura observationes. *Breslau*, 1835. Metetamata circa dentium evolutionem. *Breslau*, 1835.

and accepting a few, mentioned, in his lecture at the College of Surgeons in 1838, my researches in terms of great commendation, as having confirmed those of the continental anatomists, and in some points extended further than they. I drew up an account of my researches in a paper, entitled, "On the structure of the teeth, the vascularity of those organs, and their relation to bone," which was read before the Royal Society, June 21st, 1838, having been presented by Thomas Bell, Esq.* This was the first account written upon the structure of the teeth in this country; and in addition to the confirmation of Purkinje's and Retzius' views, it contained several new points. The most important of these was the vascularity of the tubular structure.

At a later date, in the same year, Professor Owen read a paper on the structure of the teeth before the British Association. Shortly afterwards Mr. Nasmyth published a work on the same subject, which contained a full account of Retzius' views, together with a detail of the results of his own researches.

---

### LECTURE IV.

DEVELOPMENT OF ANIMAL TISSUES FROM NUCLEATED CELLS—DEVELOPMENT OF THE TEETH FROM PAPILLÆ COMPOSED OF CELLS—THE PAPILLÆ DERIVED FROM MUCOUS MEMBRANE—THREE STAGES OF DENTITION; FOLLICULAR, SACCULAR, AND ERUPTIVE.

GENTLEMEN:—Now that we have become acquainted with the dental tissues in their perfect state, we will proceed to trace them through their various stages of development.

If we take an egg of the common fowl, and carefully in water remove first the shell, and then the albumen or white, a small circular spot of a whitish color may be seen on the surface of the yelk. This, the germinal spot, is composed of a number of spherical bodies, called cells, each having a proper coat, which

* Proceedings of the Royal Society, June 21st, 1838. LONDON MEDICAL GAZETTE, Feb. 16th, 1839; and July 10th, and July 31st, 1840. Lancet, July 25th, 1840. Dublin Medical Press, Aug. 19th, 1840.

encloses numerous lesser spherical bodies, called the nuclei. The nuclei again contain other still smaller bodies, called the nucleoli.

From these so-formed tri-celled bodies, (which by some authors are called cytoblasts, by others nucleated cells,) all the tissues of the adult animal are developed. Filling the interspaces left by the spherical cells is a colorless homogeneous fluid, described under the name of plasma. With the commencement of incubation the cytoblasts increase in size by the rapid growth of the enclosed cells, till the coat divides, and the enclosed cells are liberated. These in their turn give birth to others; the process of development being repeated until the germinal spot has greatly increased in size. At a specific time the cells begin to arrange themselves definitely for the formation of the tissues. Each tissue requires a peculiar modification of the cellular development, which, taking place gradually, may be seen at various stages of formation, from the simple nucleated cell to tissues in which the form of the elementary cell is no longer observable. Thus, in fibrous tissue, the formative cells are developed in length, and the original form lost, while in cartilage they preserve their oval figure, are placed side by side, and become separated by an intermediate tissue, which is supposed to be developed by their coats. Thus, we have growth taking place prior to the existence of vessels; not, however, without the presence of an animal fluid. This fluid, the plasma, bathes the surface of the cytoblast, and is probably furnished by the yelk.

Having given you briefly an outline of the process of development generally, we shall be prepared to examine with advantage the modification which pertains to the formation of the teeth. But our attention will be first directed to what may be termed the development of the physical form of teeth apart from the development of the individual dental tissues.

For our knowledge of this branch of the subject we are principally indebted to Mr. Goodsir; and as the matter of this lecture is in great part borrowed from his memoir, I shall follow the arrangement adopted in his description; beginning with the earliest appearance of the dental organs, and tracing their progressive development through its various stages up to their perfect state. As early as the sixth week of uterine existence, when the human embryo is scarcely an inch in length, preparation for the dental development may be seen. On opening the cavity, which can hardly at this period be called a mouth, so rudimentary is its state, a groove is found bounded anteriorly by the lips, and posteriorly, by a lobe, of semi-circular shape (the rudiment of the future palate.) This groove is called the primitive dental groove, and in it, the first stage of dental formation takes place. At the seventh week of uterine life a slight projection of the mucous membrane, at the bottom of the groove, on each side of the arch, is observable, and which soon increases in size, and forms a papilla. This papilla is the primary condition of the tooth pulp, and is composed of an aggregation of cytoblastic cells. At this early stage vessels do not go into the papilla, but form loops under it in the mucous membrane. However, as the papilla increases in size by the growth of the cytoblasts, in the manner already described, the vascular loops become elevated into its substance, always, however, lying a certain distance below the surface. This, the first formed, is the follicle of the anterior temporary molar; those of the upper appear a short time before those of the lower jaw.

About the eighth week a second follicle is formed, anterior in situation to the one first spoken of, and is accompanied by a growth in the form of a notched lamina, proceeding from the external wall of the dental groove.

At the ninth week of the fœtal life the papilla for the development of the incisive teeth make their appearance, and are bounded anteriorly by the external wall of the dental groove in the form of notched laminæ.

With the tenth week the papilla for the posterior temporary molar is developed in the dental groove posterior to those described.

Thus at the tenth week of fœtal existence we have in the dental grooves twenty papillæ, corresponding to the twenty temporary teeth. As the papillæ grow, the walls of the dental groove increase, and send out laminæ towards each other, which meeting, unite and form septa. By these means the papillæ are enclosed in follicles (cells with open mouths.)

The development of septa commences around the first molar about the tenth week, and is principally effected by the development of processes from the external wall of the dental groove. A similar follicula development takes place around the canine papillæ. During the eleventh and twelfth week septa pass from the outer to the inner side of the anterior portion of the dental groove, and in so doing enclose in well developed follicles the incisive papillæ. By a similar process the posterior molar papillæ is follicularised; still, however, leaving behind it an open portion of the dental groove.

Considerable changes ensue during the thirteenth week, espepecially in the shape of the papillæ, which instead of remaining as hitherto simple rounded blunt masses, each of them take a particular shape. The incisive assume in some degree the shape of the incisor teeth, the canines become simple cones, and the molars resemble cones flattened transversely.

The papillæ, from their more rapid growth, protrude from the mouth of the follicle, while the depth of the latter is relative to the length of the fang of the future tooth; the canine follicle therefore being the deepest.

Simultaneous with the change of shape in the papilla is the development of opercula, or lids to the follicles. The incisive follicles have two opercula; one larger, anterior, and rather external; the second smaller, posterior and internal. There are three for the canines, one external and two internal; four or five for the molars, each corresponding with a tubercle, while the edges of the opercula correspond with the fissures on the grinding surface of the tooth.

At the fourteenth week the inner lip of the dental groove has increased in size, and applies itself in a valvular manner to the outer lip.

The relative rapidity of growth between the palpillæ and the follicles is now reversed, and the former recede into the latter. The opercula, from increased size, almost hide the papillæ.

With the termination of the fourteenth week, the primitive dental groove having performed its part in the animal economy, in furnishing the ten milk papillæ, is succeeded by the secondary dental groove, which is situated on a higher level, and is destined to furnish the papillæ of the permanent teeth, excepting, however, the molars. The secondary dental groove, gradually appears in the form of a small crescent-shaped depression, immediately behind the inner opercula of each of the milk follicles.

Those of the central incisors appear first, followed by the laterals, canines, anterior bicuspides, and posterior bicuspides. About this time the opercula close the mouth of the follicles, but without adhering; shortly, however, adhesion takes place, first closing the anterior follicles, then the laterals, the others following in succession. Between the fourteenth and fifteenth week, the opercula and lips of the now extinct adherent groove become flocculent and rough, excepting, however, the depressions for the ten permanent teeth. By the adhesion of the opercula the follicles have become sacs, and the enclosed papillæ are now recognised as the pulps of the milk teeth. The crescent-formed depressions, developed in the posterior wall of the milk follicles, now constitute the secondary dental groove, or cavities of reserve, for furnishing the papillæ of the ten anterior permanent teeth; and by a process similar to that by which the milk papillæ were developed in the primitive dental groove.

It will be recollected that a posterior portion of the primitive dental groove, after the formation of the papilla of the second milk molar, remained open and unoccupied. This portion is subject to no alteration till the expiration of the sixteenth or seventeenth week, when the development of the papillæ and follicles for the anterior molars commences, the site being immediately behind the sac of the posterior milk molar. The cavities of reserve for the ten anterior permanent teeth exist, and as minute compressed sacs lying between the milk sac and the surface of the gums. From the time of the closure of the milk follicles the pulps gradually assume their peculiar shape, and those destined for the function of the molar teeth are divided at the base for the development of their several roots. With these progressive changes, the sac growing faster than the pulp, an intervening space is formed in which is developed a soft granular substance, which for a time increases in quantity, and is adherent to the inner surface of the sac, but not to the pulp, though closely applied to the surface of the latter.

In describing the course of the vessels of the pulps, I cannot do better than use Mr. Goodsir's own words.

"Each branch of the dental artery, as it arrives at the fundus of its destined sac, sends off a number of radiating twigs, which run in the substance of the cellular submucous tissue (which constitutes the outer membrane of the sac) towards the gum, from which others proceed to inosculate with them. The combined twigs then ramify minutely in the true membrane of the sac, without sending the smallest twig into the granular substance. The dental branch, after giving off these saccular twigs, divides into a number of contorted ramifications between the base of the pulp and the sac, from which smaller *ramusculi* are transmitted into the pulp itself. In the case of the molars, the main branches divide into three secondary branches, one for each of the secondary bases. From these three sets of saccular twigs, three packets of contorted pulp vessels take their origin."

After the conversion of the incisive follicles into sacs, and the change in form of the pulp, the follicle of the first permanent molar is converted into a sac, which then receives granular matter.

In the conversion of this follicle into a sac, a portion between the continuous surface of the gum and the sac remains inadherent, thus forming a second sac with collapsed sides. This, lined with mucuous membrane, is a cavity of reserve for the formation of the secondary permanent molar and the wisdom tooth. The next step in the process of the development is the appearance of caps of tooth-substance upon the tips of the pulps, and is accompanied with a diminution of the granular matter of the sacs. Ultimately the granular matter is reduced to a thin layer, and at last, when the pulp is perfectly capped with tooth-substance, disappears altogether, leaving the interior of the sac with a villous vascular appearance, like mucous membrane.

These changes bring us to the seventh or eighth month.

During the interval of the fourteenth week and eighth month, the cavities of reserve for the ten anterior permanent teeth gradually recede from their position between the milk sacs and the gums, to points posterior to the milk sacs.

About the fifth month, the distal extremities of the four anterior cavities of reserve having dilated, the rudiments of the permanent papillæ are seen in a small fold, lying across the base of the cavity, the direction corresponding with that of the cutting edge of the future tooth. At this time the cavities of reserve resemble in shape a pear, the smaller end being directed towards the surface of the gum. At the small extremity two folds appear, one anterior, the other posterior, and together round off the extremity of the sac. These folds are rudimentary opercula, and are lost with the obliteration of the smaller portion, which they cut off for the larger division of the sac. With the obliteration of the distal extremity the cavities are converted into closed sacs, and continue to recede deeper in the gum behind the temporary sacs, embedding themselves in the sub-mucous tissue of the milk sacs. We have now arrived at a stage of dental development which has often been described, but of which Dr. Blake was the first to give a distinct account. This author, finding the temporary sacs with lesser ones implanted in their posterior walls, was led to conclude that the permanent tooth pulp is formed from the temporary by a gemmiparous process. Subsequent writers, including Mr. Fox and Mr. Bell, but excepting Hunter, have, prior to the publication of Mr. Goodsir's excellent paper, held similar opinions. The more extended and accurate observations of the latter author have, however, led to the explosion of the gemmiparous hypothesis.

About the fifth month the septa are formed between the external and internal walls of the primitive dental groove, and ossification takes place in them, first forming little bridges, which, by the end of the sixth month, form complete partitions. Thus, at the termination of the sixth month of uterine life, the alveoli are formed. As the milk sacs increase in size the alveoli increase also, and little niches are formed in their posterior walls for the reception of the permanent sacs. The permanent sacs, when formed, increase in size more rapidly than the jaw, from which want of accordance the sac of the first permanent molar, at the eighth month, retreats into the maxillary tuberosity, where it is found completely embedded, and occupying a higher level than the other sacs. In thus moving backward, the cavity of reserve placed between it and the gum is dragged out to an increased length, and with it the surface of the gum is drawn upwards and backwards, thus producing a dimple on its surface. About the time of birth the fangs of the incisors begin to be formed. Three separate actions are concerned in the development, viz. first, elongation of the base of the pulp; second, deposition of dentine upon it; third, adhesion of the contiguous portion of the sac to the surface of the so-formed dentine.

When the development of the roots of the milk teeth is advancing, the permanent sacs increase in size, together with the containing crypts; which, from a growth of their edges, form an osseous partition between the temporary and permanent sacs. At this period, when the infant is eight or nine months old, the maxillary arch having increased in size, the permanent molar begins to descend from its elevated position in the tuberosity, and the cavity of reserve to resume its original position and size.

Commonly the central incisors pass through the gum (or are cut, as it is more frequently called) about the eighth or ninth month, and in the following manner: The crown of the tooth being perfected, and the formation of the fang advanced by the triplex action already described, an action is set up by which the edge of the tooth passes through the gum. Here, then, terminates the saccular stage of the tooth, the sac having been opened by the passage of the tooth through the gum. It must be borne in mind, that with the development of dentine for the fang the sac becomes adherent to its surface, not, however, to the surface of the enamel, so that a probe might be passed down the surface of the enamel to the neck of the tooth so soon as the sac is opened by the edge of the tooth. When once the tooth is cut, growth progresses rapidly. The tooth, however, appears to grow more rapidly than it really does, and from the following cause: The sac being opened, and its inner surface thereby rendered continuous with that of the gum, a strong disposition to contract seems to come into force in that portion investing the enamel, and as the gum constitutes one fixed point, and the adhesion of the sac to the neck of the other, the tooth is, as it were, lifted out of the gum by the shrinking of the sac. As a consequence of this movement, the distance between the unfinished end of the fang and the fundus of the alveoli is lengthened. The alveolus now rapidly adapts itself to the neck of the tooth, to which it becomes accurately moulded. The pulp elongates itself, and diminishes at its base, till, at the completion of a tooth, it has diminished to the size of a thread, and is occupied principally by the dental vessels and nerves.

As the temporary teeth have advanced towards the surface, the sacs for the permanent teeth have receded behind the former, and have become enclosed in a proper bony crypt, from each of which a foramina proceeds. In the sacs for the front teeth these foramina open immediately posterior to the milk teeth, but those for the bicuspids open into the alveoli of the milk molars. From the apex of each sac a fibrous cord proceeds through the foramina to join the gum near the neck of the milk teeth, excepting in those under the milk molars, in which the fibrous cord unites with the periosteal lining of the temporary alveolus. These cords, or gubernacula as they are sometimes called, are formed of the obliterated portion of the pulp follicle, which, it will be remembered, was rendered external to the sac by the development and subsequent closure of the opercula. It seems the union of the two sides is sometimes incomplete, so that the cord is in fact a tube closed at its two extremities. The gubernaculum lengthens as the sac recedes from the surface, and disappears only after the tooth passes through the gum. From observing the position and disappearance of the gubernaculum, many have supposed that it leads or directs the developing tooth to its proper situation in the alveolar arch. Mr. Goodsir makes the following observation when speaking of the use of the cords and foraminæ:—"The cords of communication which pass through these foramina are not tubular, although in some instances a portion of the unobliterated intra-follicular compartment of the original little cavity of reserve may be detected in them; they are merely those portions of the gum which originally contained the lines of adhesion of the depressions for the permanent teeth in the secondary dental groove, and which have been subsequently lengthened out in consequence of the necessarily retired position in which the permanent teeth have been developed during the active service of the temporary set.

The cords and foramina are obliterated in the child, either because the former are to become useful as "gubernacula," and the latter as "itinera dentium;" or much more probably in virtue of a law which appears to be a general one in the development of animal bodies; viz. *"that parts or organs which have once acted an important part, however atrophied they may afterwards become, yet never altogether disappear so long as they do not interfere with other parts or functions."*

The sacs of the permanent teeth derive their vessels first from the gums, but afterwards from the milk sac; and as the sac sinks deep into the alveolus they receive their vessels from the proper dental canals. When speaking of the development of the first permanent molar, it was stated that after the closure of the sac, a space or cavity lined with mucous membrane was left between the sac and the surface of the gum; also that the cavity was stretched backwards as the sac receded into the tuberosity of the jaw, and that as the sac from increase of space is placed in the line of the alveolus, the cavity again contracted and occupied its former position. This position, however, is soon changed, for at the seventh or eighth month the cavity enlarges and elongates posteriorly, dips back behind the sac for the first permanent molar, and then gives birth to another papilla; the papilla of the second permanent molar.

In the formation of this sac a cavity of reserve is, however, left, just as in the formation of the sac of the first permanent molar. This cavity is elongated, and again contracts as it is dragged by the new sac backwards towards the tuberosity; and again, as in the former case, comes forward as the sac descends into the line of the alveolus, when by the growth of the jaw sufficient alveolar room is afforded. About this period the cavity of reserve for the third time enlarges posteriorly, dips backward behind the sac of the second molar, and forms a papilla and sac for the development of the wisdom tooth. The so-formed sac of the wisdom tooth, recedes, as did the anterior molar, into the tuberosity of the maxilla. The cavity of reserve now exists but as a line, which extends through the gum over the permanent molars, marking the points where it formerly existed as an important organ of development.

At present we have been describing the dental development as it occurs in the upper jaw. In the lower maxilla the process is similar, the primitive dental groove appearing first at the side of the jaw, and afterwards advancing towards the centre. The papilla of the first milk molar is the first to appear, then that of the canine, followed by those for the incisors. In the development of the permanent molar the coronoid process there receives the sacs, as the tuberosity did those in the upper jaw. The cavities of reserve for the pemanent molars are, however, formed from an unclosed posterior portion of the secondary dental groove, and not from the primitive dental groove, as in the upper jaw. In point of time the appearance of the papillæ is a few days later in the under than in the upper jaw. Mr. Goodsir divides dentition into three stages. First, the follicular, in which stage is included the papilla, when it existed as a simple prominence from the mucous membrane, and extends to the closure of the opercula. Second, the saccular, which commences with the closure of the opercula, and ends with the passage of the tooth through the gum. Third, the eruptive, which commences when the tooth appears through the gum, and extends to the period when the permanent teeth are fully developed.

These three stages, when considered in reference to any particular tooth, are well defined, but when viewed in reference to the whole set or the two sets, they are intermingled; thus, when one tooth germ is in the saccular, another is in the follicular stage; again, when the temporary teeth are in the eruptive, the permanent teeth are in the saccular stage.

It will have been observed in the foregoing statement, that though the papillæ of the upper incisors are the first to appear, yet that the lower incisors are the first to be completed. This arises from an arrest in the development in the superior incisors, consequent upon the slow development of the anterior part of the superior maxilla. In the fœtus three lobules are found forming the central part of the upper jaw; these lobules are the rudiments of the intermaxillary bones. In the formed mouth of the human subject, the intermaxillary are merged into the maxillary bones, but in the lower animals they exist in distinct parts. These central lobules, according to Mr. Goodsir, are slow in their development, and with them the contained papillæ, while the papillæ of the lower incisors grow steadily from the time of their first appearance in the primitive dental groove. For a further account of this part of the subject, I must refer you to Mr. Goodsir's paper.

From the foregoing statement (the matter of which has been taken from Mr. Goodsir's memoir) we learn that early in fœtal life a groove appears in the mouth, between the jaw and the lips, first in the side, afterwards in the anterior part of the mouth; that in this groove small papillæ start up, first at the side of the mouth, then in the anterior part; that walls are developed from the external boundary of the groove, enclosing the papillæ in cells, and that afterwards coverings, or lids, are developed. We also learn, that from the posterior surface of the milk cells secondary cells are formed, which go through stages like those by which they have been preceded, and give birth to the permanent teeth. We further learn that the milk teeth are formed in three distinct divisions—a molar, a canine, and an incisor; that the molar is the first, the canine the second, and the incisor the third to appear; but that the first molar is developed before the second, and the first incisor before the second; also, that the rudiments of the teeth appear in the upper jaw before the lower. In the closing of the follicles the process commences at the anterior part of the dental groove, and proceeds backwards. In the permanent teeth, the papillæ, excepting the anterior molar, appear first at the mesial line, and in succession backwards.

It is thus apparent that the organs for the development both of the temporary and permanent teeth are derived from the mucous membrane of the mouth, and that the papillæ of the temporary and permanent teeth have each a perfectly independent origin.

*Diagrams illustrative of the Formation of a Temporary and its corresponding Permanent Tooth from a mucous membrane.*

FIG. 1.—Mucous membrane.

FIG. 2.—Mucous membrane with a granular mass deposited in it.

FIG. 3.—A furrow or groove on the granular mass, (primitive dental groove.)

FIG. 4.—A papilla on the floor of the groove, (a tooth germ.)

FIG. 5.—The papilla enclosed in a follicle in the bottom of the groove, (the latter in the condition of a secondary dental groove.)

FIG. 6.—The papilla acquiring the configuration of a pulp, and the sac acquiring opercula. The depression for the cavity of reserve behind the inner operculum.

FIG. 7.—The papilla becomes a pulp, and the follicle a sac, in consequence of the adhesion of the opercular lids. The secondary dental groove in the act of closing.

FIG. 8.—The secondary groove adherent, except behind the inner operculum, where it has left a shut cavity of reserve for the formation of the pulp and sac of the permanent tooth.

FIG. 9.—The last change rendered more complete by the deposition of the granular body (the enamel pulp,) deposition of tooth-substance commencing.

FIG. 10.—The cavity of reserve receding from the surface of the gum, and dilating at its distal extremity, in which a pulp is forming. Rudimentary opercula developing near its proximal extremity, and dividing it into a follicular and extra-follicular compartment.

FIG. 11.—The temporary tooth acquiring its fang by the triplex action described in the lecture, and its sac approaching the surface of the gum.

FIG. 12.—The temporary tooth sac again a follicle: free portion of the sac becoming shorter, and the fang of the tooth passing from the bottom of the socket.

FIG. 13.—The temporary tooth completed; the free portion of the sac become the vascular border of the gum; the adherent portion become periosteum of the fang. The permanent tooth sac removed from the gum, but connected with it by the gubernaculum passing through the foramen behind the temporary alveolus.

FIG. 14.—The fang of the permanent tooth lengthening, and the crown approaching the gum. The fang of the temporary tooth undergoing absorption.

FIG. 15.—The perfect permanent tooth.

### *Illustrations of the Formation of the Three Molar Teeth.*

FIG. 1.—The non-adherent portion of the primitive dental groove.

FIG. 2.—Papilla and follicle of the first permanent molar on the floor of the non-adherent portion, which is now a portion of the secondary dental groove.

FIG. 3.—The papilla and follicle of the first molar become a pulp and sac. The lips of the secondary groove adhering, so that the latter has become the posterior or great cavity of reserve.

FIG. 4.—The sac of the first molar advanced along a curved path into the substance of the coronoid process or maxillary tuberosity. The cavity of reserve lengthened out or advanced with it.

FIG. 5.—The sac of the first molar returned by the same path to its former position. The cavity of reserve again shortened.

FIG. 6.—The cavity of reserve sending backwards the sac of the second molar.

FIG. 7.—The sac of the second molar advanced along a curved path into the coronoid process or maxillary tuberosity. The cavity of reserve lengthened for the second time.

FIG. 8.—The sac of the second molar returned to the level of the dental range. The cavity of reserve shortened for the second time.

FIG. 9.—The cavity of reserve sending off the pulp and sac of the wisdom tooth.

FIG. 10.—The sac of the wisdom tooth advanced along a curved line into the maxillary tuberosity or coronoid process.

FIG. 11.—The sac of the wisdom tooth returned to the extremity of the dental range.

### LECTURE V.

### ON THE DEVELOPMENT OF THE DENTAL TISSUES.

HAVING traced the formation of the teeth as organs, it now remains for us to investigate the manner in which the several tissues comprising these organs are developed. On entering this investigation it will be necessary to bear in mind, first, that all textures are formed from cells, and that the first act of development consists in the formation of these cells, and the second in the formation of other cells from those already existing. This last is beautifully illustrated in the development of the dental organs. Thus the papillæ springing up from the primary dental groove are composed of nucleated cells, cells capable of producing, by the growth of the contained nuclei, other cells similar in character and in purpose to those from which they have been formed. The dental papillæ increase in size by the formation of new cells in the foregoing manner. In a short time the papillæ are contained in follicles; still, although much increased in size, they are composed of nucleated cells connected by a homogeneous transparent thick fluid, a blastema or plasma. In the base of the papilla, when enclosed in a follicle, blood-vessels are formed; they, however, are not immediately concerned in the cellular development, but probably, by transudation through their coats, furnish the homogeneous plasma. In process of time, the precise period varying in the different teeth, the follicles become sacs.

The papilla, which is now designated the pulp, assumes the shape of the future tooth, and it commences to fulfil the ultimate purpose of its existence, in the formation of dentine. Previous to the development of tooth-substance the inner surface of the sac becomes separated from the surface of the pulp, the intervening space being occupied by a soft gelatinous granular matter. This is the formative pulp for the development of the enamel. It is composed of characteristic nucleated cells suspended in a plasma, each being derived from the sac. At this stage of dental formation we have a closed sac containing two formative pulps, one for the development of the dentine, the other for the formation of the enamel; the former in the shape of the crown of the future tooth, the latter forming a cap over the dentine pulp. At a later period we have a matrix for the formation of the cementum.

The dentine pulp, from its earliest appearance to the time of its transition into dentine, is composed of a series of nucleated cells, united and supported by plasma, is supplied with vessels, and, during the greater part of its existence, with nerves. The cells, during the growth of the pulp, have no definite arrangement; when, however, dentine is about to be formed, they arrange themselves into lines near the surface of the pulp, first

FIG. 10.

FIG. 11.

FIG. 10.—Pulp for the development of dentine, with nucleated cells scattered through its substance.
FIG. 11.—Dentine pulp, with the cells in lines.

at its apex, afterwards at the sides. The direction of these lines of cells is vertical to the surface of the pulp, similar to the direction of the dentinal tube of a developed tooth. Those cells on and near the surface of the pulp are the larger, the relative size decreasing the further they are removed from the surface. The lesser cells, however, increase to an equal size to the larger ones, when the time for their calcification arrives. Intermediate and connecting laterally the lines of cells is a subgranular gelatinous tissue, corresponding in position to that described as the plasma, with which it is identical, but is now developed further; each cell after falling into line divides into two or more in its length, and each division elongates, (Fig. 12.) A transparent central nucleus or space is seen in each cell, which lengthens with the cell. The cells by their increased length become placed end to end, and ultimately unite; and the elongated central space of each individual, by a further development, joins and opens into those of the super-imposed cells; thus forming a central tube common to the linearly united cells. At this period of development the earthy matter is received into the cellular or rather tubular and intertubular tissue, whereby the gelatinous matrix having assumed the required form is converted into tubular and intertubular tissue; in other words, into dentine. In some instances the linearly arranged cells have two or even three central spaces, but these in the progress of development become joined in one.

FIG. 12.

FIG. 13.

FIG. 12.—Dentine pulp, with the cells placed end to end, and the nuclei elongated. Granular intercellular tissue.

FIG. 13.—Dentine pulp, with the cells united, and the nuclei developed into tubes.

Covering the pulp is a transparent membrane closely united to the external cells. This membrane, which forms the exterior of the dentine, is the first to undergo calcification. Upon the external surface of this membrane are developed the hexagonal pits for the reception of the ends of the enamel fibres. The progressive stages of development are very readily observed in the molar teeth of the hog, or in a kitten, at birth. In each of these animals I have examined the formative pulps, and from these examinations I have given this description, and have made my illustrative diagrams.

Calcification begins in the surface of the pulp, commencing at the apex or apices, and gradually extending down the sides and towards the centre. As the more external and larger cells become hardened, the inner ones increase in size, assume the linear arrangement, and they in their turn become converted, by the addition of the salts of lime, into dentine, till at last the great bulk of the pulp is transformed, leaving only a comparatively small portion, with the nerves and blood-vessels, occupying the centre of the tooth.

In the progressive development of dentine from without inwards, two of the more external unite with one of the more internal lines of cells. The two central canals also unite and form one. By the frequent repetition of this process of union between the contiguous cellular lines, as they proceed inwards and towards the centre of the pulp, while the number of tubes gradually diminish, the branches of the dentine tubes are formed, (Fig. 14.) The structure composing the walls of the cells prior to the addition of the phosphate of lime, is, as far as has at present been seen, homogeneous; further investigation will, however, probably show it to be minutely granular, and similar to the ultimate structure of the intercellular tissue of bone.

FIG. 14.

FIG. 14.—Two branch lines of cells uniting to form one trunk.

The development of the dentine or ivory has been investigated by Mr. Nasmyth, who gives the following account: "On examining the internal structure of the pulp generally, the number of minute cells presenting themselves is very remarkable; they seem, indeed, to constitute the principal portion of its bulk. These vesicles vary in size from the smallest perceptible microscopic appearance, probably the ten-thousandth of an inch in diameter, to one-eighth of an inch, and are evidently disposed in different layers throughout the body of the pulp."*

At another place, he says, "The formative surface of the pulp displays a regular cellular arrangement which I have denominated reticular, and which may be described as resembling a series of skeletons of desiccated curves."†

When speaking of the formation of the ivory from the cells, Mr. Nasmyth says, "At an early stage of dental development the reticulated or cellular appearance of the pulp is particularly beautiful.

"When merely a thin layer of ossific matter has been deposited on its surface, it may with great facility be drawn out entire, together with the former, laid on a glass, compressed, and then examined with the high powers of the microscope. The different layers of cells may be seen, and the transition into ivory may be observed."‡

From these extracts it will be seen that Nr. Nasmyth had upon this subject a clear idea, to which he gave distinct expression: first, that the pulp is composed of cells, and secondly, that the dentine or ivory is formed of these cells by the imbibition of earthy matter. A few months subsequent to the appearance of Mr. Nasmyth's paper, Mr. Owen wrote a paper on the development of the teeth, in which he advanced views in the main similar to those originated by Mr. Nasmyth. Mr. Owen, however, in his work, "Odontography," gives a more distinct account, and goes much further into the subject of the development of the dental tissues, than does Mr. Nasmyth in his published memoir.

Mr. Owen gives the following account of the formation of dentine. The pulp is described as being composed of nucleated cells suspended and united by a sub-granular plasma. The

* Memoir on the development and organization of the dental tissues, by Alex. Nasmyth, August, 1836, page 39.

† Page 42.

‡ Page 46.

cells are more numerous near the surface of the pulp, where they are arranged in lines vertical to its external surface. At or near the surface the nucleus of each cell divides in two in its length, thus forming two cells; each of the so-formed cells has a nucleus of its own. These cells become again divided, but in the second instance the division is transverse. Thus, within a parent or primary cell, four lesser contained cells are formed by the division of its nucleus. The primary cells may, however, give origin to a far greater number of secondary cells than four. The primary cells being placed end to end become confluent, as do the secondary cells. The nuclei of the secondary cells also increase in length, and join those of the cells situated at either end in the same line.

Neither the primary nor secondary cells are, however, arranged in a perfectly straight line, but in their union describe slight curves; the primary corresponding with the primary, and the secondary with the secondary curves of the dentinal tube. When the pulp cells have adopted the linear position, become confluent, and the nuclei arranged in continuous lines, they receive the calcareous salts, and are thus converted into the tubular element of dentine. The nuclei of the secondary cells are described as receiving at the time of the calcification of the cells a subgranular matter, rendering them opaque. With the imbibition of the earthy salts the confluence of the cells becomes perfect, and the filled nuclei become so united as to form continuous filled tubes. At the same time the intercellular (not intertubular) substance receives the hardening salts, and thus the pulp becomes perfect dentine. The description of which I have endeavored to give a slight outline is in itself very complete. Those who would become acquainted with it in detail, I must refer to Mr. Owen's work. For myself I have been enabled to confirm some of the opinions advanced. I have been unable to satisfy myself of the invariable existence of the primary and secondary cells, and of the latter being permanently contained in the former. Then, again, in a few instances I have seen appearances that might lead to the adoption of the quoted opinion. Then, again, I am by no means satisfied that amorphous subgranular salts are deposited in the tubes in any case, much less in all.

I think that I shall be able to show you that the pulp is a modification of cartilage, modified to suit its peculiar position and function, and that the development of dentine is in every way similar to the development of bone. These points must, however, be left till we have completed our investigation of the dentinal formation. You will recollect that I described the more external portion of the dentine of the fang as made of cells in a granular base. This is the first formed, and here the cells of the pulp, instead of increasing in length, widen, and the nucleus, or cavity, increases in size; it does not unite with any other, but maintains, when calcified, its permanent cellular condition. These cells may, therefore, be considered as the elements of tubes arrested in their development. Some of the cells have, however, branching tubes proceeding from their circumference; in these instances they have assumed the form of bone corpuscles. At the periphery of the dentine the intertubular matter is comparatively abundant, and highly granular.

Mr. Nasmyth considers the dentinal tubes to be baccated fibres, and not tubes. It is very obvious how the baccated appearance arises, namely, from the individual pulp-cells, which have, by their reunion, contributed to form the tube, preserving the convexity of their centres, and at the same time preserving a constriction at the point of junction—an appearance constantly seen in the process of development prior to the perfect confluence and calcification of the cells; so that the baccated appearance may be regarded as an arrest of development. The intertubular tissue Mr. Nasmyth describes as composed of cells of characteristic form. In the human subject, he says, they are somewhat square, and occupy, in single lines, the intervening space between the fibres. From his description, they may be compared to square bricks, which are imbricated and closely united, as in a building; could the fibres, or what we consider tubes, be withdrawn, we should have remaining a series of tubes, which, for illustration, we may compare with chimnies built of brick-shaped cells. I am unable from my own observation to confirm these views of dental structure.

I will now, for the sake of comparing the two tissues, osseous and dental, and their relations in development, describe to you shortly the structure of temporary cartilage, and the formation of bone. Early in the development of the embryo a series of transparent oval cells, with central nuclei, arrange themselves side by side.

In the process of growth the individual cells become separated by the interposition of a subgranular matter. Thus we have formed temporary cartilage, consisting of nucleated cells called cartilage corpuscles, embedded in a dense elastic uniting medium. This is analogous to the pulp of the tooth, the main point of difference being that the uniting medium, or plasma, of the latter is soft and gelatinous, while in the former it is dense. When the temporary cartilage is about to be converted into bone, the cells arrange themselves into lines, not, however, by separate individuals moving from one part to another, but by the development of cells from parent cells in linear series, and then at right angles to the surface, where ossification is proceeding. Here, then, we have a process exactly similar to the arrangement in lines of the cells of the pulp prior to calcification. Immediately prior to ossification, the cartilage-cells increase in size, and the central nucleus becomes granular, and appears as though enclosed in a distinct cavity. At this formative stage we have enlarged cells arranged in lines, with an intermediate tissue separating each cell from its fellow, and also the lines or columns of cells from each other. In this intercellular tissue we find the counterpart of the intercellular tissue of the dental pulp, and in each of these the calcareous salts are received, and at the same time the tissue becomes granular. At this stage the cartilage cells uniting by their ends, have the opposed walls absorbed, and are thus formed into tubes, which is the rudimentary condition of the Haversian canals. During these changes the parietes of the cells receive ossific matter, and in the process become, like the intercellular tissue, minutely granular; each cartilage corpuscle has a central nucleus, or cell, containing one or more spherules or granules; these become more distinct as the cell enlarges, but when the cells have, by their union, formed tubes, the granular contents of the nuclei are lost. Possibly they adhere to the walls of the cells, and are concerned in the development of the bone corpuscles. Around the circumference of the tubes, developed in the manner I have described, corpuscles, or bone-cells, appear, and with them their radiating tubes, the latter being directed towards the tube or canal, which ultimately becomes an Haversian canal. In comparing the development of dentine with that of bone, we find that at first sight the pulp-cavity of the former bears a very striking resemblance to an Haversian canal, and that the dentine resembles an Haversian system. On considering the subject more carefully, we find, also, that the pulp-cavity of the tooth is analogous to the medullary cavity of bone, and that the dental tubuli are analogous to the canals formed by the confluence of the cartilage cells prior to the existence of vessels in newly-formed osseous tissue. Thus it would seem that where a tubular tissue is required, the tubes are formed by a linear confluence of cells. The nuclei and the opposed surfaces disappear, thereby leaving a tube; these tubes, whether examined in osseous or dental tissues, in their earliest stage of formation, have circular indentations both on the external and internal surfaces, marking the lines of junction of the individual cell, but as development proceeds, disappear. And further, it would appear that the so-formed tubes may either retain their original character, or may become canals for vessels. This observation is equally applicable either to bone or vascular dentine. It should, however, be recollected, in making the comparison, that the cells of temporary cartilage immediately before ossification are much larger than those of the dental pulp at a similar period.

### THE ENAMEL PULP.

Subsequent to the closure of the opercula of the tooth follicles, a granular matter is described by Mr. Goodsir as occupying the space between the internal surface of the dental sac and the surface of the pulp. This the enamel-pulp, like the dentine-pulp, is composed of peculiar and characteristic cells.

It will be remembered that the external surface of the dentine is formed of a dense transparent structure, indented on the surface with hexagonal depressions, (Fig. 15.) Upon this the enamel rests. The external surface of the dental sac is composed of submucous membrane; while the internal surface to which the enamel-pulp is adhering is formed of a transparent homogeneous membrane, divided on its internal surface with hexagonal cells or depressions, (Fig. 16.) Into these depressions the cells of

FIG. 15.

FIG. 16.

FIG. 15.—External coronal surface of the dentine.
FIG. 16.—Basement membrane of the enamel pulp.

the enamel pulp are received, and from which they proceed in columns toward the surface of the dentine, where they terminate in the hexagonal pits. The cells composing the enamel pulp are larger than those of the dentine pulp, are more transparent, and the nuclei less distinct, clear, and without granules. In the columns they are strongly united. The lateral union between these columns is slight. If the tooth sac be carefully opened at a favorable period, the enamel pulp adheres to the inner surface of the sac, and under the microscope presents, when placed in fluid, a purulent appearance, produced by the columns of cells, the free extremities of which float about like the villi of the small intestine of the carnivorous animals. Each column is seen to be attached to a hexagonal depression, and while the cellular composition of the columns is very distinct the membrane to which they are fixed seems structureless, resembling the basement tissue of mucous membrane. Upon the external surface of this, which might be called the basement tissue of the enamel pulps, vessels ramify.

FIG. 17.

FIG. 17.—Enamel pulp; *a*, basement membrane; *b*, lines of cells; *c*, vascular layer external to the basement membrane.

I may here again call your attention to the fact illustrated in the subject before us, that the development of tissues goes on without the immediate presence of vessels. In making the comparison with mucous membrane, I should perhaps remind you that the latter is composed of two separate parts; namely, of a transparent basement tissue and of an imposed layer of epithelial cells, which are not only placed upon, but are probably formed by, the basement tissue. In mucous membrane the cells from the external protective surface are constantly wearing off and being replaced by others. In the enamel pulp they receive earthy matter and become extremely hard, form a thick coat, and are to the more organized dentine what the epithelial cells are to the mucous membrane; differing, however, from the former in durability. This modification of the mucous membrane is extremely interesting, for you must not forget that the inner surface of the sac has been formed by a duplicature of mucous membrane. After the enamel is perfected, the tooth passes through the gum, and the mucous membrane, on again forming part of the free surface, resumes its original structure and function. For an excellent account of the structure of mucous membrane I would refer you to an article, written by Mr. Bowman, in the Cyclopædia of Anatomy and Physiology.

But to return to our subject: the cells being formed in lines, they eventually become confluent; the points of union being sometimes transverse, and at other times oblique.

FIG. 18.

FIG. 19.

**FIG. 18.—*a, b, c, d,* fibres of enamel in progressive stages of development from cells.**

**FIG. 19.—Enamel in the last stage of development prior to the disappearance of the line of union between elements composing the fibres.**

At this stage the earthy elements are received, and the lines of union between the component cells of the fibres become less distinct, and are eventually lost, leaving a continuous fibre (Fig. 19.) The nuclei, from the first very small, are altogether lost in the formation of the fibres, or exist as very fine tubes passing through the length of each fibre. The lateral union between the fibres is at this stage very slight, so that a fragment of newly formed enamel may with great readiness be reduced to powder. The powder so obtained is, by the aid of the microscope, seen to be composed of enamel fibres disunited laterally. Even when a tooth passes through the gum the lateral union is not very strong. We see a proof of this in the broken surface produced by the pressure of the forceps on teeth that have from want of sufficient alveolar space been removed soon after their appearance. The enamel, however, continues for a considerable time to increase in density, and at length becomes so hard that it is with difficulty that a steel instrument is made to produce any effect upon its surface. Recently developed enamel, from the innumerable interspaces between the fibres, is very opaque or pearly. These interspaces, however, become gradually less numerous, and at last, in perfectly formed enamel, are almost entirely lost. Thus we have what may be termed a progressive growth towards the perfecting of the tissue in the enamel after the appearance of the tooth through the gum; a fact which has, I think, been overlooked, especially by those who have considered that the dental tissues are devoid of vitality.

*The cementum pulp and its ossification.*—You will remember it was stated in a previous lecture, that with the commencement of the formation of the fang of the tooth, the capsules became adherent to its surface. The adhesion is effected between the inner surface of the cement pulp and the external surface of the dentine of the neck and fang; the development of the fang portion of the dentinal pulp and the cemental pulp being consentaneous.

The enamel pulp and the dentine pulp attain the full size of the parts for the development of which they are destined, before calcification of their substance commences. Not so, however, with the cement pulp; this increases on the external surface as calcification is progressing from within outwards. The former supplying and keeping slightly in advance of the latter.

The cement pulp is composed of nucleated cells, extended through a granular base, and each is gradually lost in the more external fibrous tissue of the coat of the dental sac. The nucleated cells, as regards general character and use, are analogous to those of the other dental pulps, but in appearance more closely resemble the nucleated cells of temporary cartilage. They are oval in shape, have their long axes placed transversely, and at right angles to the length of the tooth. When so arranged, the nuclei being transparent, present the appearance of cavities, which, indeed, they ultimately become. The parietes of the cells, and the interposed granular tissue, receive the phosphate and other salts of lime. The cells, with the intervening blastema nearest the surface of the dentine, are the first to be ossified; and the action commencing on the inner is gradually extended towards the outer surface of the cement pulp till the whole is ossified. Near the neck of the tooth, where the cement pulp exists only as a thin layer investing the dentine, blastema is alone present, but as the layer thickens progressively towards the end of the root, the nucleated cells appear. If we examine a single cell, or corpuscle, as these bodies are commonly called, we shall find that at its first appearance it is small, with one or two nuclei; that it gradually increases in size; the nuclei also increasing, are succeeded by a cavity; that the walls of the cell, with the surrounding blastema, receive the salts of lime; and that, lastly, the radiating tubes are developed. The cemental pulp when first developed seems to have entangled in its substance some of the fibres of the enclosing sac. Whether these fibres are ultimately removed or become identified with the blastema remains to be decided. In development, the cemental pulp bears a very close analogy to the progressive formation of cartilage; for the development of the flat bones, in which, at the various stages of growth, we find a thin border of cartilage in which the osseous tissue is developing. This border of cartilage increases in the outer surface while it decreases by conversion into bone in the inner surface.

The osseous layer of the fang is very liable to hypertrophy, which state we are accustomed to speak of as exostosis. In these cases an action is set up whereby new cemental pulp is formed, and transformed into cement. The action is accompanied by inflammatory symptoms, and resembles in its results that action which produces thickening of bone, which also is generally ascribed to inflammation. As dental exostosis constitutes a disease, we shall come to the subject in a future lecture.

I have now given you a slight account of what may be termed the new views upon dental development, most of which have arisen from microscopic investigation. I will now occupy your time for a few minutes in describing the old views. In doing so I shall not quote the various authors who have written upon dental development, or their peculiar views. Such a course would be out of place in these lectures; but I will give you an outline of the views which were generally held.

Nothing definite was known of the stage of dental formation prior to what we now recognize as the saccular stage. The sac was described as consisting of two lamellæ, each vascular, and connected to the enclosed pulp only at its base. The pulp was stated to be covered by an extremely delicate vascular membrane, supposed to be derived from the periosteum of the jaw. At the fourth month the tops of the pulp were found coated with tooth substance, which was regarded as a secretion for the external membrane of the pulp, and lying in contact with the inner membrane of the sac.

The tooth itself was supposed to be composed of a series of superimposed laminæ, secreted by the membrane of the pulp, which, becoming exhausted by its action, progressively diminished in bulk, and gave place to the secreted substance. At length the secretion of tooth substance ceases, and then a cavity is left which is lined by its proper membrane; which I presume was supposed to be that which has been described as the secreting organ. This membrane of the pulp is, I doubt not, that which I have described to you as the first to undergo ossification; and when so changed, forms the base for the reception of the enamel fibres. In the formation of the enamel the inner membrane of the sac was supposed to be solely concerned; this was described as becoming more vascular, thickening in substance, and after undergoing these preparatory changes, pours out a thick fluid, which consolidates, and afterwards becomes hard by a process resembling crystallization. The external lamina of the sac was considered to be the organ for the secretion of the cementum.

Much has been written upon the membranes of the teeth: Mr. Bell divides these into deciduous and persistent. The deciduous membranes are two only—the two comprising the sac. The persistent membranes are three in number: first, the lining membrane of the dental cavity and the secreting membrane of tooth substance; second, the periosteum of the root; and third, the periosteum of the alveoli. The periosteum of the maxillary bones was considered as the origin of the three persistent membranes. These views, which for many years were received as established, are now superseded by those advanced by Mr. Goodsir, and which, I believe, have been confirmed by subsequent investigation made by other anatomists.

In a former lecture, when tracing the analogy between the teeth and the mandibles of birds and tortoises, I mentioned that there was considerable similarity in the mode of development. Mr. Owen has found that the bill of the tortoise is developed in separate parts by papillæ of the teeth. The parts at first separate, become confluent as the process of formation advances; so that at birth the papillary origin is no longer traceable.

## Figures and Tables

**Figure f1:**
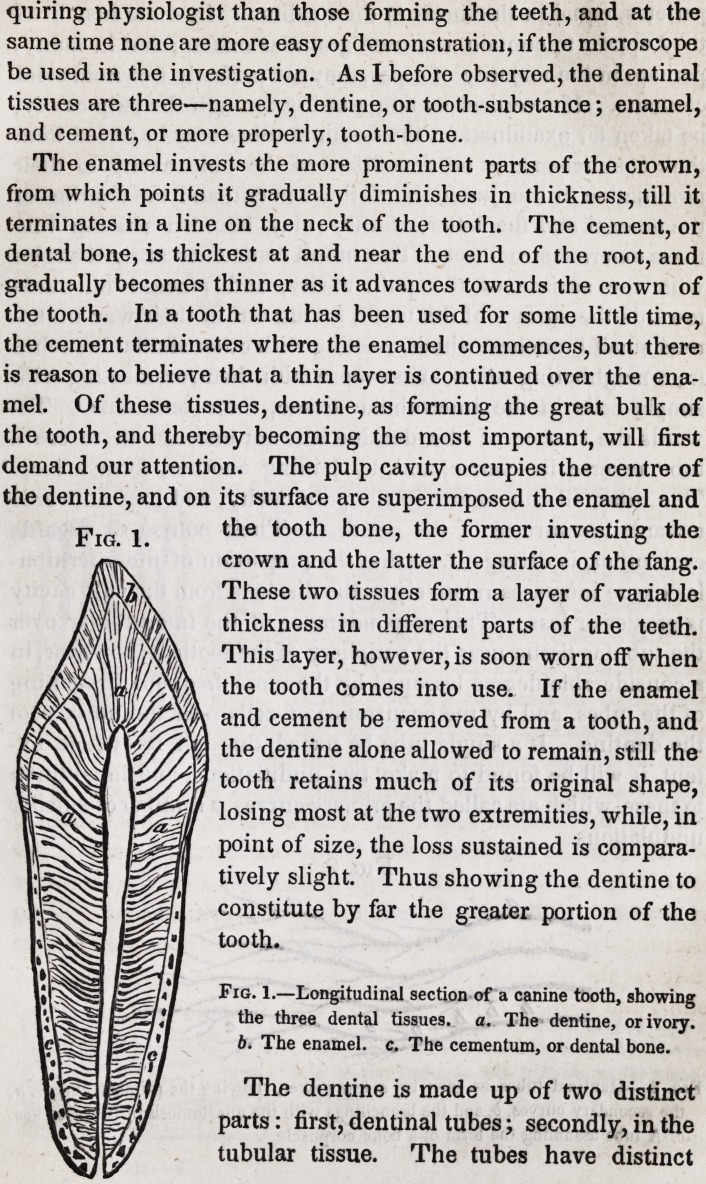


**Fig. 2. f2:**
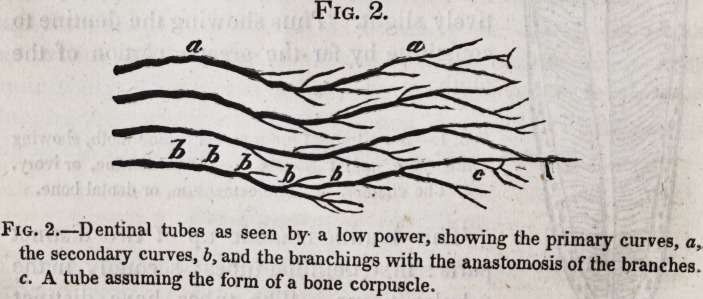


**Figure f3:**
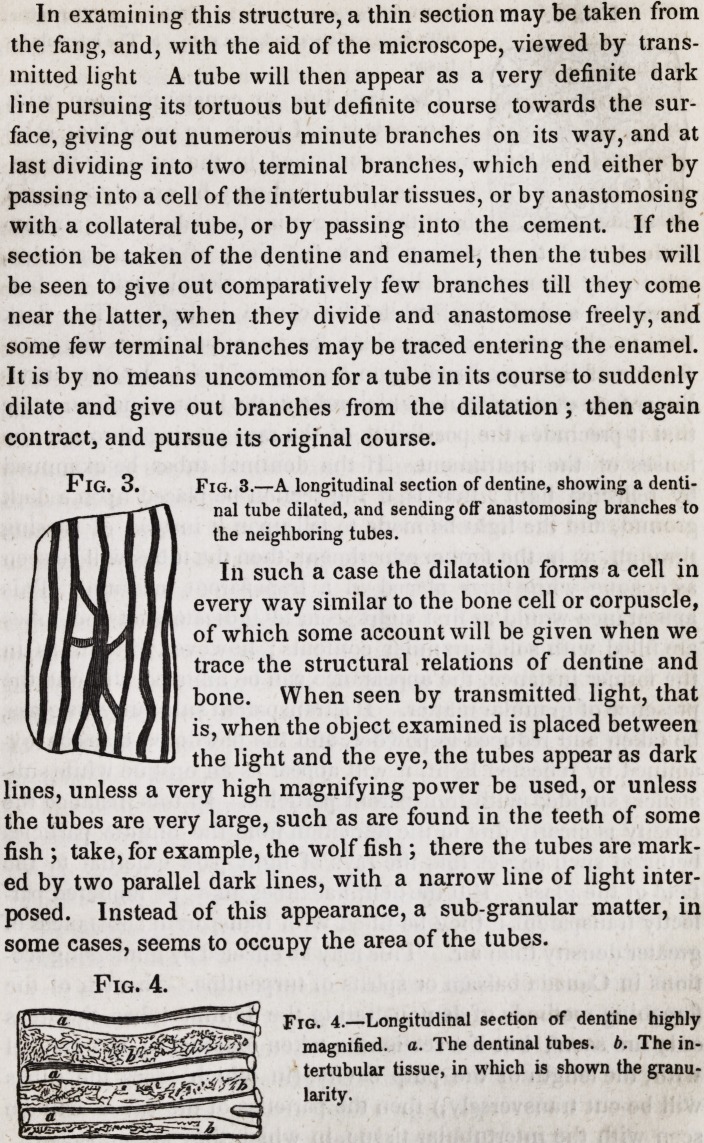


**Figure f4:**
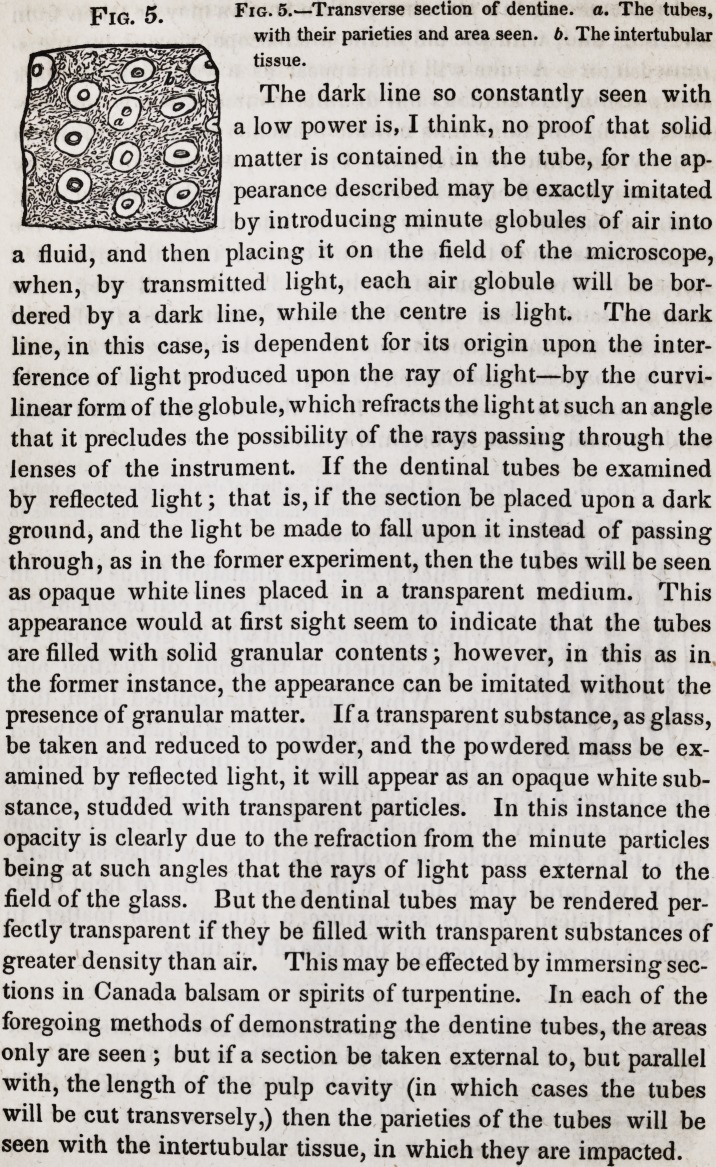


**Fig. 6. f5:**
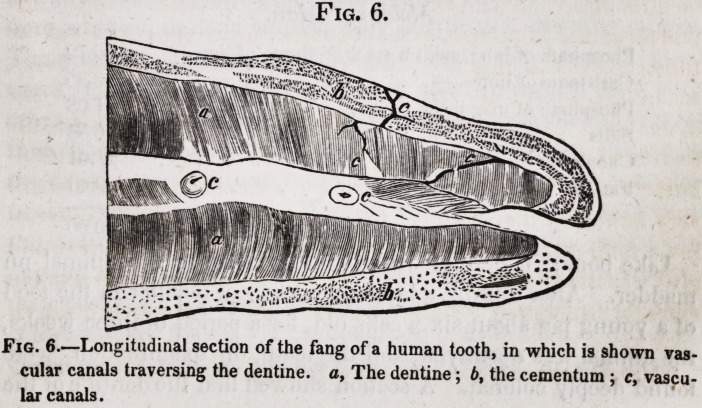


**Fig. 7. f6:**
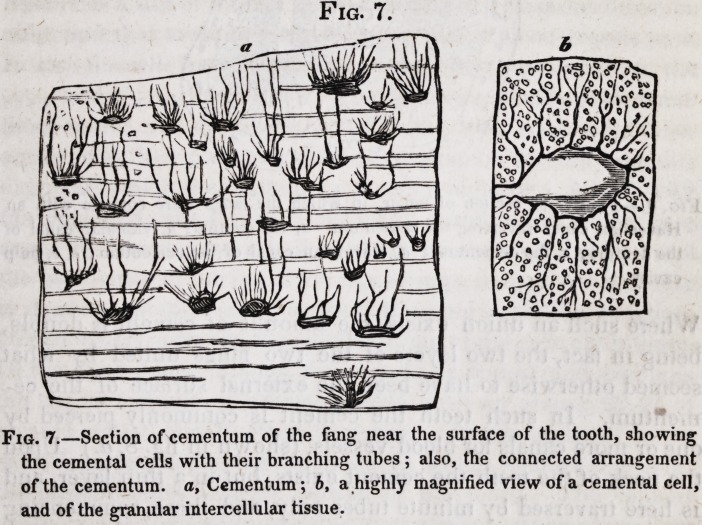


**Fig. 8. f7:**
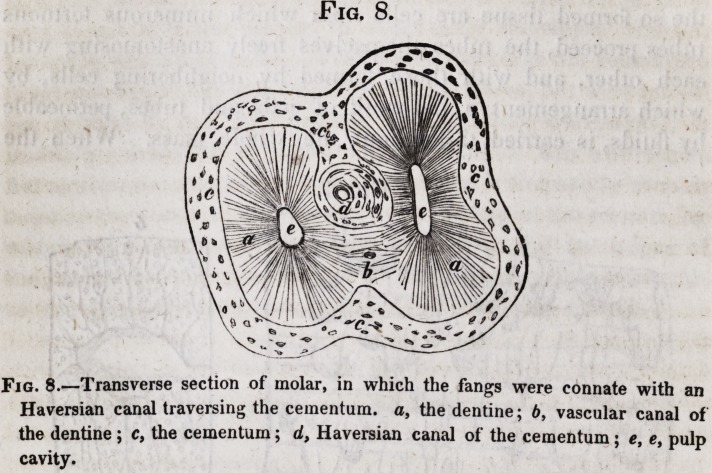


**Figure f8:**
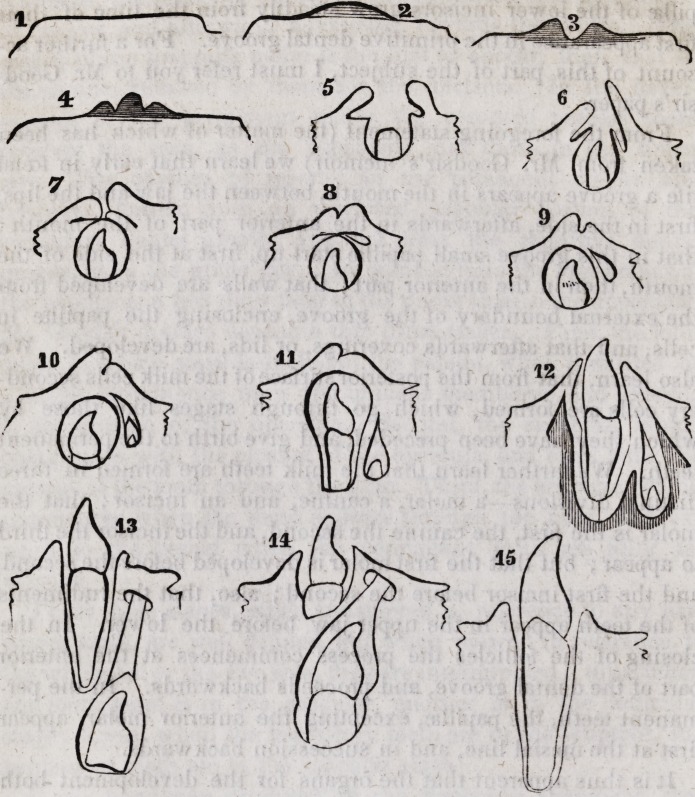


**Figure f9:**
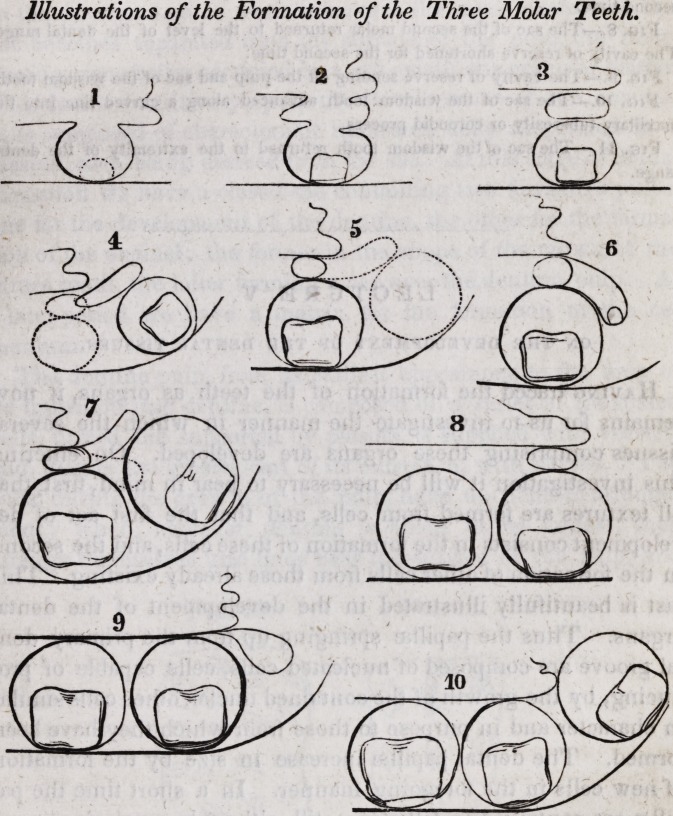


**Figure f10:**
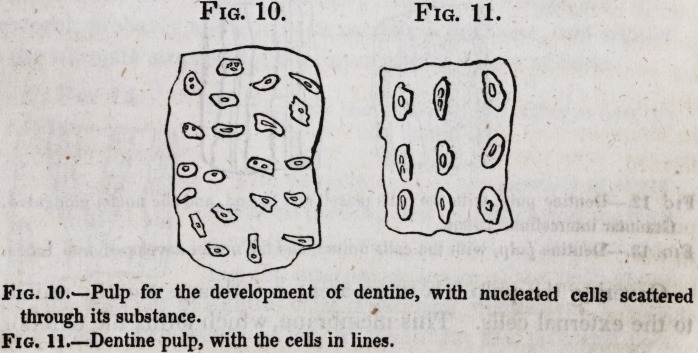


**Figure f11:**
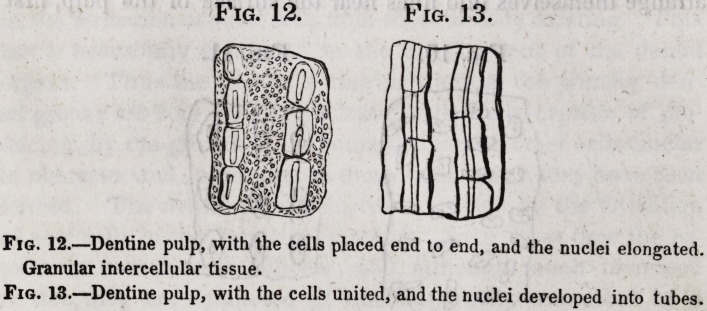


**Figure f12:**
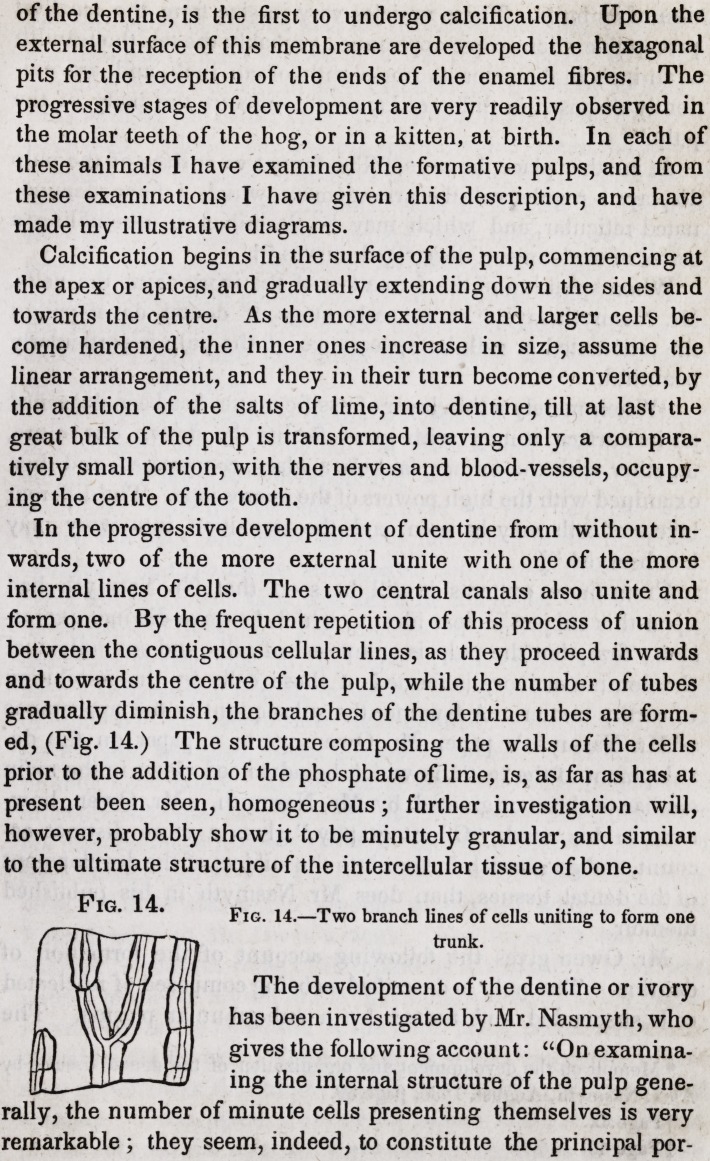


**Figure f13:**
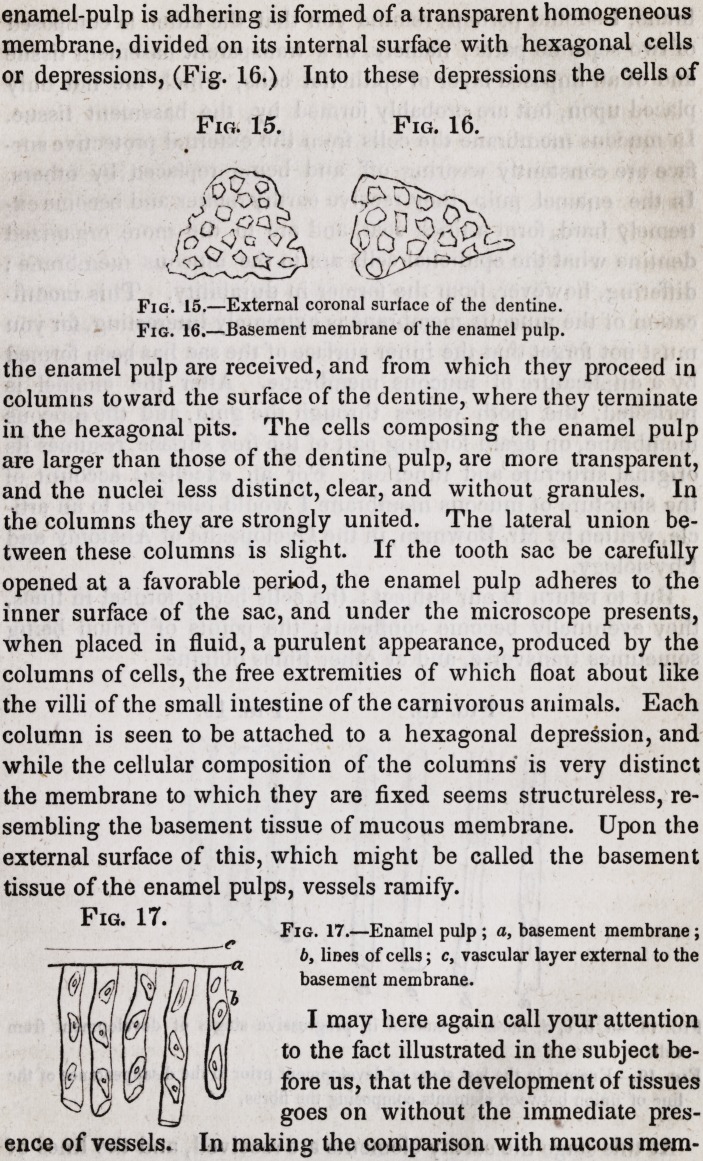


**Figure f14:**